# BINOL-Based Zirconium Metal–Organic Cages: Self-Assembly, Guest Complexation, Aggregation-Induced Emission, and Circularly Polarized Luminescence

**DOI:** 10.3390/nano16020132

**Published:** 2026-01-19

**Authors:** Yawei Liu, Gen Li, Roy Lavendomme, En-Qing Gao, Dawei Zhang

**Affiliations:** 1 State Key Laboratory of Petroleum Molecular & Process Engineering, Shanghai Key Laboratory of Green Chemistry and Chemical Processes, School of Chemistry and Molecular Engineering, East China Normal University, Shanghai 200062, China; 52264300075@stu.ecnu.edu.cn; 2 Chongqing Key Laboratory of Precision Optics, Chongqing Institute of East China Normal University, Chongqing 401120, China; 3 Laboratoire de Chimie Organique & Laboratoire de Résonance Magnétique Nucléaire Haute Résolution, Université libre de Bruxelles (ULB), Avenue F. D. Roosevelt 50, CP160/06, B-1050 Brussels, Belgium; roy.lavendomme@ulb.be

**Keywords:** metal–organic cages, chiral materials, host–guest chemistry, aggregation-induced emission, circularly polarized luminescence

## Abstract

The development of nanoscale chiral materials with enhanced optical properties holds significant promise for advancing technologies in light-emitting devices and enantioselective sensing. Here, we report the self-assembly of chiral metal–organic cages from an axially chiral, AIE-active binaphthyl dicarboxylate ligand. This supramolecular architecture functions as a multifunctional platform, demonstrating a high affinity for anionic guests through synergistic electrostatic and hydrogen-bonding interactions. The rigid cage framework not only enhances the ligand’s intrinsic aggregation-induced emission (AIE) but also serves as a highly effective chiral amplifier. Notably, MOCs significantly boost the circularly polarized luminescence (CPL), achieving a luminescence dissymmetry factor (|g_lum_|) of 1.2 × 10^−3^. This value represents an approximately five-fold enhancement over that of the unassembled ligand. The photophysical properties of this chiral supramolecular system provide a strategic blueprint for designing next-generation optical nanomaterials.

## 1. Introduction

Metal–organic cages (MOCs), formed through the coordination-driven self-assembly of organic ligands and metal ions, have emerged as a prominent class of supramolecular architectures due to their structurally well-defined cavities and synthetic tunability [1,2,3,4,5,6,7,8,9]. These discrete molecular entities are far more than static containers; they function as dynamic nanoscale platforms capable of molecular recognition [10,11,12,13], catalysis [1,14,15,16,17], separation [18,19,20], and the stabilization of reactive species [21]. The intrinsic programmability of MOCs allows for the precise spatial organization of functional moieties, which is a key requirement for developing cooperative systems with emergent properties. A particularly compelling frontier in this field is the construction of enantiopure, chiral MOCs. Such structures can transfer and amplify inherent chirality, offering immense potential for applications in enantioselective recognition/sensing, asymmetric catalysis, and advanced chiroptical materials [5,22,23,24,25,26].

Circularly polarized luminescence (CPL), which refers to the emission of chiral light, is a highly desirable property for cutting-edge technologies including quantum information processing, ultra-sensitive probes, and next-generation 3D displays [27,28]. The integration of CPL activity into the well-defined structures of MOCs is, therefore, a sought-after goal. However, achieving high-performance MOC-based CPL materials is challenging [24,25,29,30]. Many MOCs are inherently non-fluorescent or weakly fluorescent. This is often due to quenching mechanisms such as photoinduced electron transfer or the population of low-lying metal-to-ligand charge transfer (MLCT) and ligand-to-metal charge transfer (LMCT) states, which provide efficient non-radiative decay pathways for the excited state. Another significant issue is the aggregation-caused quenching (ACQ) effect, where the emission of conventional chromophores is quenched in the aggregated or solid state—precisely the conditions required for practical device applications [25,31,32]. Consequently, constructing MOCs with strong CPL activity is difficult: it requires not only a robust and well-defined chiral environment but also a framework designed to generate intense and stable light emission.

A powerful strategy to overcome ACQ to achieve fluorescent MOCs is the use of aggregation-induced emission luminogens (AIEgens) [24,33]. AIE-active molecules exhibit weak emission in solution but become highly emissive in the aggregated state due to the restriction of intramolecular motions (RIMs) [34]. The construction of MOCs from AIEgenic linkers has thus been explored to create highly emissive nanomaterials [35,36]. Logically, designing MOCs from building blocks that are inherently both chiral and AIE-active represents a promising strategy to generate strong CPL. In such an integrated system, the rigid, preorganized supramolecular framework can simultaneously enforce the chiral configuration and restrict molecular motion, thereby amplifying both the emission intensity and the chiroptical response. In this regard, binaphthyl is a well-known axially chiral building block with a high racemization barrier and inherent photoluminescence properties. It has previously been shown to afford AIEgens and CPL emitters [27,37,38,39,40]. The binaphthyl scaffold has been widely employed to impart chirality in the construction of MOCs [41,42,43]. Nevertheless, the deliberate exploitation of its intrinsic AIE characteristic within these chiral cages to achieve and amplify CPL activity remains rare [24].

Our group has recently developed a synthetic strategy to access soluble MOCs based on zirconium, a metal known for forming highly stable Cp_3_Zr_3_O(OH)_3_ (Cp = *η*^5^-C_5_H_5_) clusters [18,19,44]. By employing the bulky, weakly coordinating anion tetrakis(3,5-bis(trifluoromethyl)phenyl)borate (BAr_F_^−^), we minimize cation–anion interactions during self-assembly, thereby conferring excellent solubility to cationic Zr-MOCs and facilitating their study and application in solution [18,19]. Based on this method, we herein report the design and self-assembly of a new class of chiral zirconium metal–organic cages. We employ a rationally designed, axially chiral BINOL (1,1′-bi-2-naphthol)-derived ligand that intrinsically incorporates AIE activity, anion-binding pyridinium groups, and carboxylate coordination sites. The resulting MOC serves as a multifunctional supramolecular platform that demonstrates (i) strong and selective binding of anionic guests via synergistic electrostatic and hydrogen-bonding interactions; (ii) enhanced aggregation-induced emission (AIE) due to the rigid cage framework restricting intramolecular motion more readily than the free ligand; and (iii) significantly amplifies circularly polarized luminescence (CPL) in the solid state, with a luminescence dissymmetry factor (g_lum_) five times greater than that of the building block.

## 2. Materials and Methods

**Materials.** All the chemicals and solvents used for synthesis and analysis were purchased from commercial sources and used without further purification. (4-Methoxycarbonylphenyl)(mesityl)-iodonium tetrafluoroborate (compound **C**) was synthesized according to the literature [45]. Aqueous solutions in all experiments were prepared using deionized water.

**Methods.** NMR spectra were recorded using a Bruker 400 MHz Avance III HD Smart Probe (^1^H, ^13^C, ^19^F, and 2D experiments). Chemical shifts for ^1^H, ^13^C and ^19^F are reported in ppm on the *δ* scale. High-resolution mass spectra were collected on an ESI-Q-TOF MS spectrometer in a methanol solution. UV-vis absorption spectroscopy was recorded on a HIMADZU UV-2700 spectrometer in the 200–800 nm region. Fluorescence spectra were measured using the RF-6000 (Shimadzu, Japan). Circular dichroism (CD) spectra were measured on a Chirascan Series Spectrometer (Applied Photophysics Ltd., UK) at room temperature. Circularly polarized luminescence (CPL) spectra were measured using a JASCO J-1500-150 spectrophotometer at room temperature (λ_exc_ = 365 nm). All the CPL data were obtained by scanning three cycles. Scanning electron microscope (SEM) images were measured using Phenom Nano SEM. Dynamic light scattering (DLS) was measured on ZEN3700 NanoZSE. Fluorescence quantum yield (QY) and fluorescent lifetime (FL) were measured using a HORIBA FluoroCube-JY.

## 3. Results and Discussion

### 3.1. Design, Synthesis and Characterization

**Figure 1:**
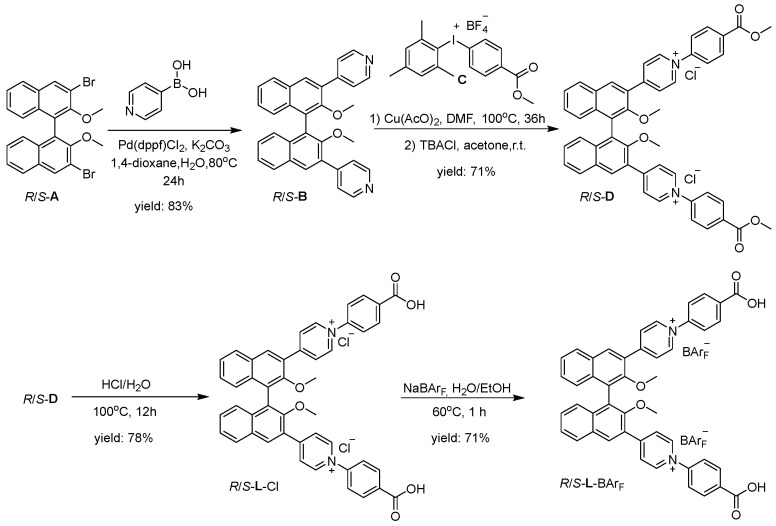
Synthesis of enantiopure *R*/*S*-**L**-BAr_F_.

The enantiopure binaphthyl-based dipyridinium-dicarboxylic ligands (*R*/*S*-**L**-BAr_F_) were prepared as BAr_F_^−^ salts in four steps (Figure 1 and Figures S1–S19). First, a Suzuki cross-coupling of *R*/*S*-3,3′-dibromo-2,2′-dimethoxy-1,1′-binaphthalene (*R*/*S*-**A**) with pyridin-4-ylboronic acid afforded the dipyridyl compounds *R*/*S*-**B**. The subsequent N-quaternization reaction of *R*/*S*-**B** with diaryliodonium salt **C** via a copper-mediated coupling yielded the dicarboxylic esters *R*/*S*-**D**, isolated as chloride salts via precipitation. The dicarboxylic acid groups were then unveiled by acid hydrolysis of the ester precursors, providing *R*/*S*-**L**-Cl. Finally, anion exchange with NaBAr_F_ in a water/ethanol mixture furnished the desired *R*/*S*-**L**-BAr_F_ as pure solids.

**Figure 2:**
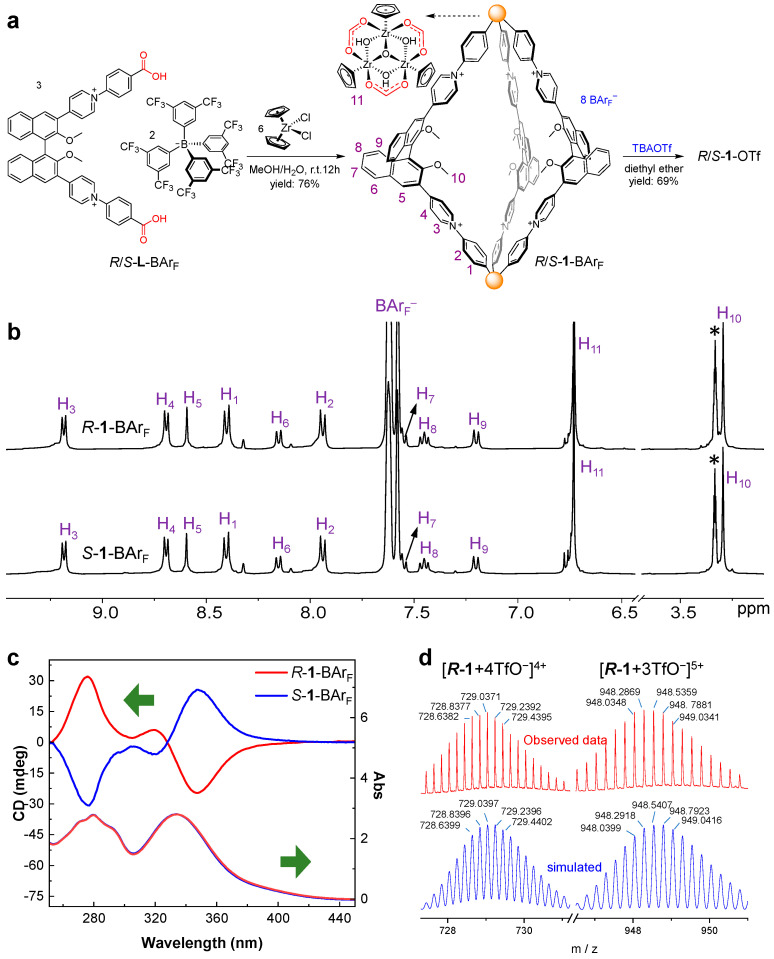
(**a**) Self-assembly of *R*/*S*-**1**-BAr_F_ and synthesis of *R*/*S*-**1**-OTf through an anion exchange strategy. (**b**) ^1^H NMR spectra (400 MHz, 298 K) of *R*-**1**-BAr_F_ and *S*-**1**-BAr_F_ in CD_3_OD. The peak of the solvent is indicated by an asterisk. (**c**) CD and UV-vis spectra of *R*-**1**-BAr_F_ and *S*-**1**-BAr_F_ in CH_3_OH. (**d**) High-resolution ESI-MS analysis of *R*-**1**-OTf.

We then investigated the self-assembly of the enantiopure Zr-based metal–organic cages (*R*/*S*-**1**, Figure 2a) following our previous method [44]. A stoichiometric reaction of *R*/*S*-**L**-BAr_F_ (1 equiv) with Cp_2_ZrCl_2_ (2 equiv) in a CH_3_OH-H_2_O mixture at room temperature for 12 h afforded a yellow solution. Subsequent addition of water into this solution prompted the precipitation of enantiopure *R*/*S*-**1**-BAr_F_. The counter anion can be easily exchanged to trifluoromethanesulfonate (TfO^−^) by adding the tetrabutylammonium (TBA^+^) salt to *R*/*S*-**1**-BAr_F_ in methanol to give enantiopure *R*/*S*-**1**-OTf (Figure 2a and Figures S31–S34).

The ^1^H NMR spectra of *R*-**1**-BAr_F_ and *S*-**1**-BAr_F_ are almost identical and display a single set of signals for both the ligand and the Cp_3_Zr_3_O(OH)_3_ cluster, consistent with the *C*_3_-symmetry of the helicate structure (Figure 2b and Figures S20–S26). The presence of the proton signals of Cp_3_Zr_3_O(OH)_3_ and the variation in the ligand signals of *R*/*S*-**1**-BAr_F_ from those of *R*/*S*-**L**-BAr_F_ support the formation of the metal–organic cages, the integral ratio being consistent with the expected 2:3 between the trinuclear cluster and the dicarboxylate ligand. All proton signals of **1** were assigned through two-dimensional (2D) NMR experiments (Figures S27 and S28). The result of the DOSY spectrum of *R*-**1**-BAr_F_ showed a diffusion coefficient of 4.7 × 10^−6^ cm^2^/s, and the effective diameter of the helicate cage is calculated to be 1.6 nm using the Stokes–Einstein equation (Figure S29). The low-resolution ESI-MS spectrum of *R*-**1**-BAr_F_ shows a series of peaks corresponding to the cationic species [C_2_L_3_^8+^ + xBAr_F_^−^]^(8−x)+^ (x = 1 − 4) (Figure S30), evidencing the successful assembly of the helicate cage. The identity of the C_2_L_3_-type cage (C = cluster {Cp_3_Zr_3_(μ_3_-O)(μ_2_-OH)_3_} and L = ligand) was further confirmed by high-resolution electrospray ionization mass spectrometry (ESI-MS), which presents molecular ions of [**1** + 3OTf]^5+^ and [**1** + 4OTf]^4+^ for both *R*-**1**-OTf (Figure 2d) and *S*-**1**-OTf (Figure S35).

**Figure 3:**
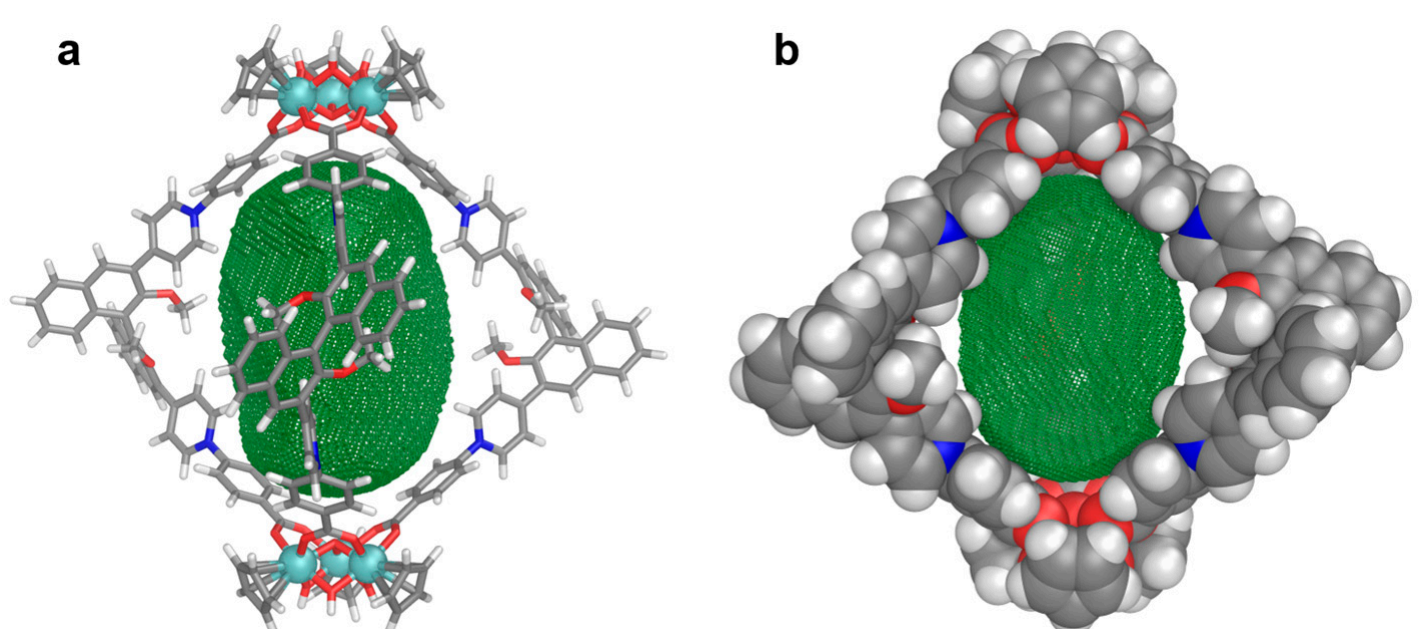
PM7-optimized model of *S*-**1** shown in stick (**a**) and space-fill (**b**) modes. The 708 Å^3^ cavity defined by a probe of radius 5.1 Å is shown in green mesh.

The structure of **1** was optimized at the PM7 level of theory [46] using the program MOPAC (version 22.0.5) [47] with the default PM7 parameters (for details, see Section S6 in the Supplementary Materials). As shown in Figure 3, *S*-**1** has a cage-like framework with approximate *D*_3_-symmetry. The cavity shown in green mesh is surrounded by three ligands, giving a cavity volume of 708 Å^3^ (probe radius: 5.1 Å) calculated using the MoloVol program [48]. The distance of *μ*_3_-O···*μ*_3_-O between the two vertices is about 19.6 Å, comparable to the result of the DOSY experiment.

The circular dichroism (CD) spectra of the enantiomeric *R*-**1**-BAr_F_ and *S*-**1**-BAr_F_ display mirror-image signals in methanol from 260 to 450 nm, each exhibiting characteristic split-type Cotton effects (Figure 2c). With respect to the lowest energy transition, *R*-**1**-BAr_F_ and *S*-**1**-BAr_F,_ respectively, exhibit negative and positive Cotton effects at 346 nm. The CD spectra of *R*-**1**-BAr_F_ and *S*-**1**-BAr_F_ are similar to those of the corresponding *R*-**L**-BAr_F_ and *S*-**L**-BAr_F_ (Figure S36), with minor differences in the region from 290 to 325 nm. The UV spectral profiles of *R*/*S*-**1**-BAr_F_ and *R*/*S*-**L**-BAr_F_ are largely the same (Figure 2c and Figure S36), with two primary bands at 280 nm and 335 nm. Both the cage (*R*/*S*-**1**-BAr_F_) and the ligand (*R*/*S*-**L**-BAr_F_) are nearly nonfluorescent in methanol (see below).

### 3.2. Guest Binding Studies

The BAr_F_^−^ anion has a weak binding capability toward the coordination cage, owing to its bulky volume, low charge density, and trifluoromethyl substituents [49,50]. We thus employed **1**-BAr_F_, instead of **1**-OTf, as the host to investigate the guest-binding properties of the cage. The very weak interactions of BAr_F_^−^ with **1** were further confirmed by titrating a large excess of NaBAr_F_ into a methanol solution of *R*-**1**-OTf, showing no noticeable shifts for the proton signals of *R*-**1**-OTf (Figure S37).

**Figure 4:**
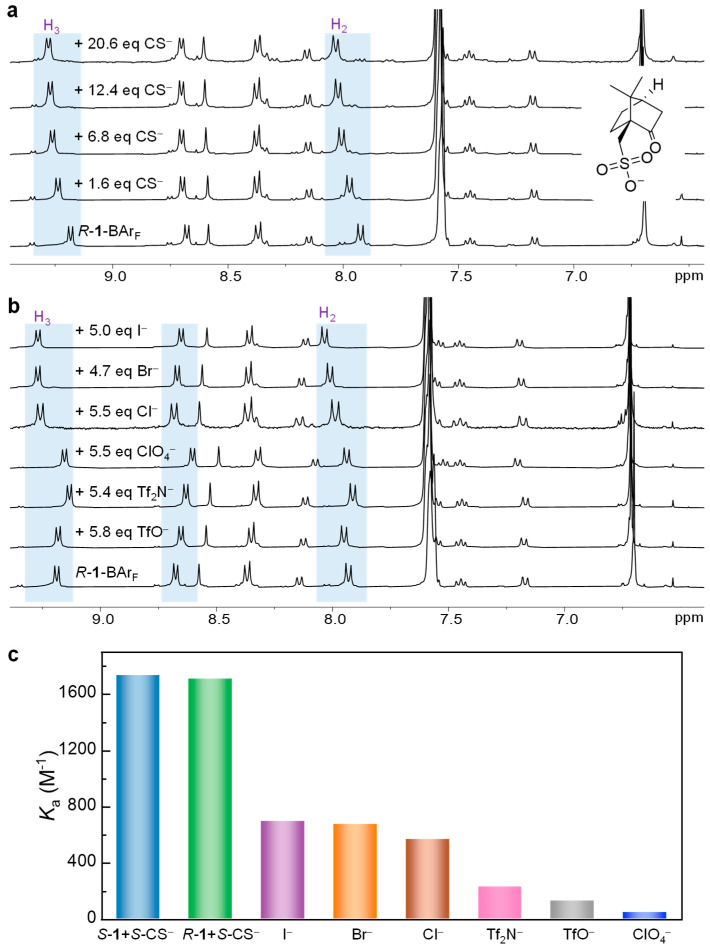
(**a**) ^1^H NMR (CD_3_OD, 400 MHz, 298 K) titrations of *R*-**1**-BAr_F_ (0.3 mM) with *S*-CS^−^. (**b**) ^1^H NMR (CD_3_OD, 400 MHz, 298 K) spectra of *R*-**1**-BAr_F_ (0.3 mM) in the absence or presence of TfO^−^, Tf_2_N^−^, ClO_4_^−^, Cl^−^, Br^−^, or I^−^. (**c**) Binding constants (M^−1^) of **1** for various anions in CD_3_OD at 298 K.

Owing to the intrinsic axial chirality imparted by the binaphthyl skeleton of the cage, we first explored the host–guest interactions of **1** with the chiral camphorsulfonate anion (CS^−^). As shown in Figure 4a and Figure S38, titration of *S*-CS^−^ into a solution of *R*-**1**-BAr_F_ in CD_3_OD resulted in gradual shifts in the proton signals of the cage in the ^1^H NMR spectra, particularly for protons H_2_ and H_3_, which obviously moved downfield. On the other hand, the peaks of CS^−^ experienced a significant upfield shift compared to the signals of free CS^−^ (Figure S38). Therefore, during titration of *R*-**1**-BAr_F_ with CS^−^, the CS^−^ signals presented gradual downfield shifts to approach the signal of free CS^−^. These observations are consistent with the shielding effects of **1** on the bound guest and indicate a binding event that is fast on the NMR timescale. The titration data analysis with BindFit [51] suggests that the binding occurs in a 1:1 stoichiometry with a binding affinity of 1708 ± 182 M^−1^ (Figure 4c and Figure S38). We also investigated the interaction between *S*-CS^−^ and *S*-**1**-BAr_F_ (Figure S39) and found that the binding constant of *S*-**1**-BAr_F_ for *S*-CS^−^ (1736 ± 171 M^−1^) was very similar to that of *R*-**1**-BAr_F_ for *S*-CS^−^. The result suggests that *R*/*S*-**1**-BAr_F_ does not exhibit enantioselective recognition properties for CS^−^, presumably due to the separation of the chiral and binding sites in the very open pocket of **1** (Figure 3). Our previous work has established that the ortho-protons on the pyridinium moiety are acidic and can serve as hydrogen bond donors for binding sulfonate anions [19]. We therefore attribute the significant chemical shift changes in both H_2_ and H_3_ to the binding of *S*-CS^−^, facilitated by a combination of electrostatic and hydrogen-bonding interactions. This assumption has been further confirmed by computational results of the host–guest model, showing the C–H···O hydrogen bonds between H_2_ in **1** and the sulfonate group in CS^−^ (Figure S54). The binding event around the achiral pyridinium moiety is hardly influenced by the chirality of the binaphthyl moiety, giving no noticeable enantioselective recognition for CS^−^.

The guest binding properties of **1** for achiral anions (Cl^−^, Br^−^, I^−^, TfO^−^, ClO_4_^−^, and Tf_2_N^−^, as tetrabutylammonium salts) were investigated using *R*-**1**-BAr_F_. ^1^H NMR titrations with the various anions resulted in gradual shifts in the host resonances (Figure 4b and Figures S40–S45), indicating fast-exchange binding on the NMR timescale. Notably, distinct chemical shift patterns were observed. The halide anions induced significant downfield shifts for H_2_ and H_3_, whereas other anions generally caused weak upfield shifts for these protons. The corresponding 1:1 binding constants were quantified using BindFit, showing the following affinity hierarchy: I^−^ > Br^−^ > Cl^−^ > Tf_2_N^−^ > TfO^−^ > ClO_4_^−^ (Figure 4c). We infer that the preference of host **1** for halide anions, especially for I^−^, likely stems from favorable interactions between the electron-deficient host and the electron-donating character of these anions—a property that is notably weaker in the cases of Tf_2_N^−^, TfO^−^, and ClO_4_^−^. Moreover, the observed affinity trend I^−^ > Br^−^ > Cl^−^ aligns with their relative electron-donating abilities, further supporting this explanation [44]. Note that when the solvents were switched from pure methanol to MeOD/H_2_O = 9:1, the binding constants remained largely unchanged (Figures S46 and S47), indicating a negligible effect of water on anion complexation. A higher percentage of water in methanol resulted in the precipitation of the host (0.3 mM). Additionally, the binding constants of **1** for these anions in methanol were found to be too small to cause significant changes in the UV-Vis spectrum of *R*-**1**-BAr_F_ upon titrating the anions (Figure S48).

### 3.3. Aggregation-Induced Emission

**Figure 5:**
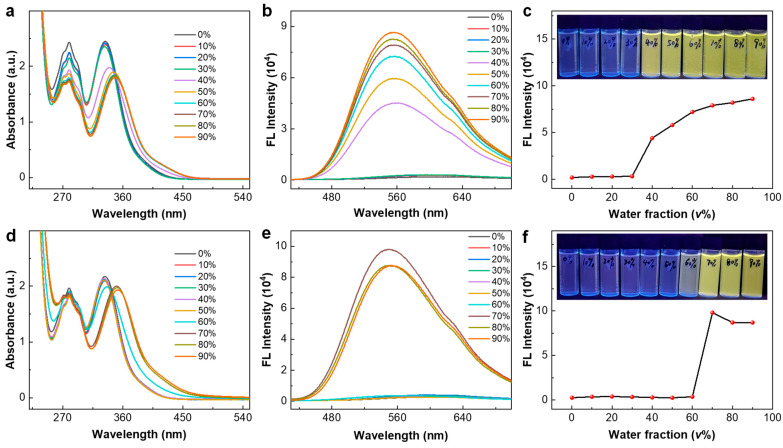
UV–vis absorption spectra (**a**), fluorescence emission spectra (λ_exc_ = 365 nm) (**b**), and fluorescence peak intensity (**c**) of *R*-**1**-BAr_F_ in MeOH-H_2_O mixtures with varying water fractions. Inset of Figure 5c: photographs of the solution at varying water fractions excited at 365 nm. UV–vis absorption spectra (**d**), fluorescence emission spectra (λ_exc_ = 365 nm) (**e**), and fluorescence peak intensity (**f**) of *R*-**L**-BAr_F_ in MeOH-H_2_O mixtures with varying water fractions. Inset of Figure 5f: photographs of the solution at varying water fractions excited at 365 nm.

The aggregation-dependent photophysical properties of *R*-**1**-BAr_F_ were investigated in MeOH-H_2_O mixtures with varying water fractions (*f*_w_) from 0 to 90%. As shown in Figure 5a, the UV-Vis absorption spectrum of *R*-**1**-BAr_F_ (12.4 µM) remained largely unchanged at *f*_w_ ≤ 30%. However, when *f*_w_ ≥ 40%, the absorption peaks at 281 and 335 nm began to decrease abruptly, with the latter peak exhibiting a redshift along with a more abrupt drop in absorbance. These spectral changes were accompanied by the formation of aggregates, leading to turbid suspensions. At the same time, an abrupt transition from the nonfluorescent state to a strongly fluorescent state was observed at *f*_w_ = 40%. The fluorescent state exhibits yellow emission, corresponding to a broad emission band at 553 nm (excited at 365 nm) (Figure 5b,c). The intensity increases with further increase in *f*_w_. The phenomena unambiguously confirm aggregation-induced absorption and emission behaviors. The fluorescence quantum yield (Φ_F_) for the aggregates of *R*-**1**-BAr_F_ was determined to be 10.6% in a MeOH-H_2_O (2:8) mixture and the excited state lifetime was measured to be 9.86 ns (Figure S50). We infer that the restriction of intramolecular motions (RIM) in the aggregation state decreases the energy gaps between lowest unoccupied and highest occupied molecular orbitals for enhanced interannular coplanarity and meanwhile suppresses nonradiative deactivation of the excited states through the motions, thus leading to redshifted absorption and promoted radiative transition [34,52,53].

We then investigated the AIE behavior of ligand *R*-**L**-BAr_F_ under the same solvent conditions for comparison with that of *R*-**1**-BAr_F_. The concentration of *R*-**L**-BAr_F_ was set at 37.2 µM in methanol, which is three times higher than that of *R*-**1**-BAr_F_ (12.4 µM) due to the presence of three ligands in one cage. The UV-Vis spectrum of *R*-**L**-BAr_F_ in methanol shows two bands at 280 and 331 nm (Figure 5d), similar to the spectrum of the cage. The free ligand also shows aggregation-induced absorption and emission behaviors in MeOH-H_2_O mixtures, featuring a notable redshift of the absorption band at 331 nm and an abrupt fluorescence turn-on at 549 nm (Figure 5e,f). However, the transition occurs at *f*_w_ ≥ 70%, which is significantly higher than that observed for *R*-**1**-BAr_F_. These results demonstrate that the MOC aggregates more readily than the ligand, exhibiting a lower aggregation threshold of water fraction, presumably due to the spatial preorganization of BINOL moieties within the skeleton of **1**. The fluorescence quantum yield (Φ_F_) for the aggregates of *R*-**L**-BAr_F_ was determined to be 8.5% in a MeOH-H_2_O (2:8) mixture, with an excited state lifetime of 9.38 ns (Figure S50). The slight enhancement of the quantum yield of the *R*-**1**-BAr_F_ aggregates compared to that of the *R*-**L**-BAr_F_ aggregates may be attributed to the more effective restriction of intramolecular motion within the cage. We also studied the aggregation-dependent photophysical properties of the *S*-cage and the *S*-ligand in MeOH-H_2_O mixtures with varying water fractions and observed nearly the same results as the *R* configuration (Figure S49).

**Figure 6:**
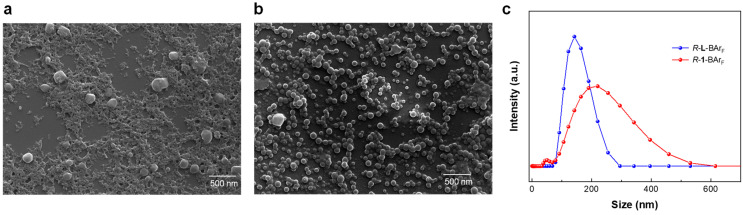
SEM images of the aggregates of *R*-**1**-BAr_F_ (**a**) and *R*-**L**-BAr_F_ (**b**) in MeOH-H_2_O (2:8) mixtures. (**c**) DLS of the aggregates of *R*-**1**-BAr_F_ and *R*-**L**-BAr_F_ in MeOH-H_2_O (2:8) mixtures.

To gain further insights, the morphologies of the *R*-**L**-BAr_F_ and *R*-**1**-BAr_F_ aggregates formed in a MeOH-H_2_O (2:8) mixture were characterized using a scanning electron microscope (SEM). The results showed the presence of regular spherical particles in the dry state for both types of aggregates (Figure 6a,b), although flocculent precipitates were also observed in the case of *R*-**1**-BAr_F_. The nanoparticles aggregated by *R*-**L**-BAr_F_ had diameters ranging from 50 to 150 nm, while those formed by *R*-**1**-BAr_F_ exhibited larger sizes, ranging from 100 to 250 nm. We also conducted dynamic light scattering (DLS) measurements on samples of *R*-**L**-BAr_F_ and *R*-**1**-BAr_F_ in MeOH-H_2_O (2:8) mixtures (Figure 6c), which confirmed the presence of spherical nanoparticles dispersed in solutions with sizes comparable to those in the dry state observed by SEM.

As the cage binds TfO^−^, we decided to investigate the effect of guest complexation on the AIE behavior of **1**-BAr_F_ by introducing 8 equivalents of TBAOTf relative to the cage. The results showed that the UV–visible and fluorescence spectral changes (Figure S51) were very similar to those of *R*-**1**-BAr_F_ in the absence of any guests (Figure 5a). The fluorescence quantum yield (Φ_F_) and the fluorescence lifetime for the aggregates of *R*-**1**-BAr_F_ in the presence of TfO^−^ in MeOH-H_2_O (2:8) were measured to be 10.0% and 10.6 ns, respectively (Figure S50). The ^1^H and ^19^F NMR spectra were used to analyze the precipitates, revealing the presence of 3 TfO^−^ among the 8 counter anions of **1**, with the other five being BAr_F_^−^ (Figures S52 and S53). These results demonstrate that the complexed TfO^−^ did not significantly perturb the AIE behavior of *R*-**1**-BAr_F_.

### 3.4. Circularly Polarized Luminescence

**Figure 7:**
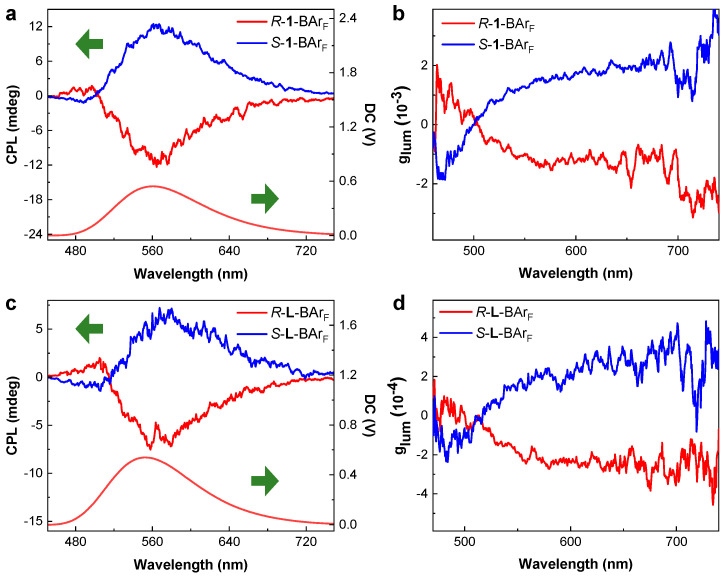
(**a**) Solid-state CPL and fluorescence spectra of *R*-**1**-BAr_F_ and *S*-**1**-BAr_F_ (λ_exc_ = 365 nm, 20 °C). (**b**) The g_lum_ spectra of *R*-**1**-BAr_F_ and *S*-**1**-BAr_F_. (**c**) Solid-state CPL and fluorescence spectra of *R*-**L**-BAr_F_ and *S*-**L**-BAr_F_ (λ_exc_ = 365 nm, 20 °C). (**d**) The g_lum_ spectra of *R*-**L**-BAr_F_ and *S*-**L**-BAr_F_. For details of the measurements, see Section S5 in the Supplementary Materials.

The incorporation of chiral binaphthyl units into the enantiopure *R*/*S*-**1**, along with their AIE behavior, inspired us to develop the cage as a promising platform for CPL-active nanomaterials [54]. Considering the very weak fluorescence of **1**-BAr_F_ in solution and the convenience of CPL measurements for solid-state samples, we measured the CPL spectra of the metal–organic cages in the solid state, using a mass ratio of potassium bromide to *R*/*S*-**1**-BAr_F_ of 5:1. As shown in Figure 7a, due to the AIE characteristics of the cages, the emission intensity of solid *R*/*S*-**1**-BAr_F_ was significantly enhanced, and mirror-image CPL signals for *R*-**1**-BAr_F_ and *S*-**1**-BAr_F_ were detected, with the dominant CPL signals appearing at 560 nm. Moreover, the luminescence dissymmetry factor (|g_lum_|), calculated as g_lum_ = 2(*I*^L^ − *I*^R^)/(*I*^L^ + *I*^R^) [55], where *I*^L^ and *I*^R^ correspond to the intensities of left- and right-handed circularly polarized light emitted by the chiral luminophores, reached a value of 1.2 × 10^−3^ (Figure 7b).

We also performed the CPL measurements for the ligands, *R*-**L**-BAr_F_ and *S*-**L**-BAr_F_ (Figure 7c,d). The results showed that similar mirror-image CPL signals were observed for *R*-**L**-BAr_F_ and *S*-**L**-BAr_F_, with consistent correlations between signal sign and chiral configuration, indicating that the CPL characteristics of the cage primarily originate from the chiral properties of the ligand. Differently, the |g_lum_| value of the metal–organic cage was measured to be five times larger than that of the ligand (2.1 × 10^−4^). This suggests that the coordination cage acts as a chiral amplifier by providing a rigid, preorganized, and symmetric scaffold that enhances both emission and dissymmetry through restricted molecular motion and optimized chiral alignment.

## 4. Conclusions

In summary, we have synthesized a pair of enantiopure zirconium metal–organic cages that function as an integrated supramolecular platform, synergistically combining anion recognition, aggregation-induced emission, and circularly polarized luminescence. These cages demonstrate selective binding toward anionic guests through a synergistic combination of electrostatic and hydrogen-bonding interactions. The rigid cage architecture significantly enhances the intrinsic AIE behavior of the ligand by restricting intramolecular motion, leading to pronounced fluorescence emission at lower aggregation thresholds compared to the free ligand. Most notably, these cages act as a powerful chiral amplifier, boosting the circularly polarized luminescence activity and achieving a luminescence dissymmetry factor five times greater than that of the molecular building block. This work demonstrates that the precise spatial organization of chiral AIEgens within a metal–organic cage provides an effective blueprint for designing advanced chiroptical nanomaterials, with promising potential for applications in enantioselective sensing, quantum information processing, and next-generation 3D displays.

## Data Availability

The raw data supporting the conclusions of this article will be made available by the authors on request.

## Supplementary Materials

The following supporting information can be downloaded at https://www.mdpi.com/article/10.3390/nano16020132/s1. Synthesis and characterization of *R*/*S*-**L**-BAr_F_, *R*/*S*-**1**-BAr_F_, and *R*/*S*-**1**-OTf (Figures S1–S36), anionic guest structures studied and their binding constants with host *R*-**1**-BAr_F_ (Table S1), host–guest binding data obtained from _1_H NMR titration studies (Figures S37–S47), UV-vis spectral titration results (Figure S48), aggregation-induced emission studies (Figures S49–S51), _1_H NMR spectra of the aggregates (Figures S52 and S53), PM7-optimized structure of the host–guest complex (Figure S54).

## Abbreviations

The following abbreviations are used in this manuscript:

BINOL	1,1′-binaphthyl-2,2′-diol
MOCs	metal–organic cages
AIE	aggregation-induced emission
AIEgens	aggregation-induced emission luminogens
ACQ	aggregation-caused quenching
CS^−^	camphorsulfonate anion
CD	circular dichroism
CPL	circularly polarized luminescence
