# Supplementary material for: BINOL-Based Zirconium Metal–Organic Cages: Self-Assembly, Guest Complexation, Aggregation-Induced Emission, and Circularly Polarized Luminescence
Source: Nanomaterials (Basel). 2026 Jan 19;16(2):132. doi: 10.3390/nano16020132 (PMC12844199; doi:10.3390/nano16020132)
Supplement: Supplementary file 1 [file nanomaterials-16-00132-s001.zip › nanomaterials-4042825-supplementary.pdf]

nanomaterials

Supplementary Materials

BINOL-Based Zirconium Metal-Organic Cages: Self-Assembly, Guest Complexation, Aggregation-Induced Emission, and Circularly Polarized Luminescence

Yawei Liu, Gen Li,* Roy Lavendomme, En-Qing Gao,* and Dawei Zhang*

Table of Contents

1. Synthesis and characterization 2
    1.1 Synthesis and characterization of *R*/*S*-**L**-BAr_F 2
    1.2 Synthesis and characterization of *R*/*S*-**1**-BAr_F 11
    1.3 Synthesis and characterization of *R*/*S*-**1**-OTf 16
2. Guest binding studies 19
3. Aggregation-induced emission 31
4. Morphology characterization 34
5. Circularly polarized luminescence 35
6. Computational calculations 36
    6.1 Structure optimization 36
    6.2 Volume calculations 36
7. References 37

1. Synthesis and characterization

1.1 Synthesis and characterization of *R*/*S*-L-BAr$_F$

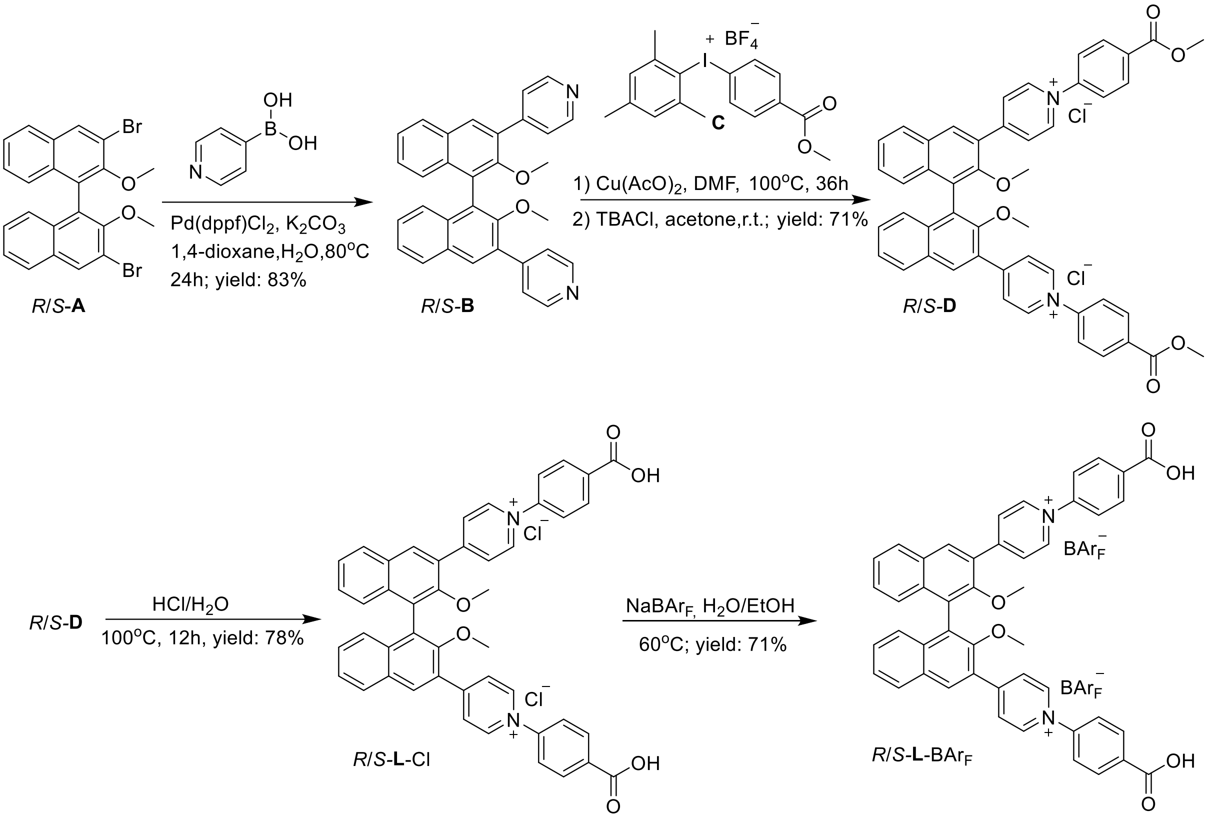

Figure S1. Synthesis of *R*/*S*-**L**-BAr$_F$.

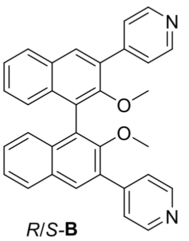

R/*S*-**B:** In a 250 mL round bottom flask, *R*/*S*-**A** (500 mg; 1.07 mmol), pyridin-4-ylboronic acid (525 mg; 4.28 mmol), Pd(dppf)Cl$_2$ (155 mg; 0.21 mmol), and K_2CO_3 (1.76 g; 12.8 mmol) were dissolved in a mixture of 1,4-dioxane (25 mL) and H_2O (5 mL). The mixture was heated at 100 °C for 24 h under nitrogen atmosphere. After cooling to room temperature, the resulting mixture was extracted with ethyl acetate. The combined organic extracts were washed with brine, dried over sodium sulfate and then concentrated under reduced pressure. The crude solid was purified by column chromatography on silica gel (hexanes/ethyl acetate, 1:1 v/v) to afford *R*/*S*-**B** as a white solid (410 mg, 83% yield).

^{1}H NMR (*R*-**B**) (CD_3OD, 298 K, 400 MHz): δ 8.66 – 8.54 (m, 4H), 8.14 (s, 2H), 8.00 (d, *J* = 8.2 Hz, 2H), 7.86 – 7.77 (m, 4H), 7.48 – 7.38 (m, 2H), 7.32 – 7.23 (m, 2H), 7.13 (d, *J* = 8.6 Hz, 2H), 3.13 (s, 6H) ppm. **^{13}C NMR** (*R*-**B**) (CD_3OD, 298 K, 100 MHz): δ 153.4, 148.9, 147.7, 134.2, 131.7, 130.9, 130.8, 128.4, 127.1, 125.9, 125.4, 125.2, 124.4, 59.9 ppm.

^{1}H NMR (*S*-**B**) (CD_3OD, 298 K, 400 MHz): δ 8.62 (d, *J* = 5.4 Hz, 4H), 8.16 (s, 2H), 8.02 (d, *J* = 8.2 Hz, 2H), 7.83 (d, *J* = 5.8 Hz, 4H), 7.45 (t, *J* = 7.5 Hz, 2H), 7.30 (t, *J* = 7.6 Hz, 2H), 7.14 (d, *J* = 8.5 Hz, 2H), 3.15 (s, 6H) ppm. **^{13}C NMR** (*S*-**B**) (CD_3OD, 298 K, 100 MHz): δ 153.4, 148.9, 147.8, 134.2, 131.8, 131.0, 130.9, 128.4, 127.0, 125.9, 125.3, 125.2, 124.4, 59.9 ppm.

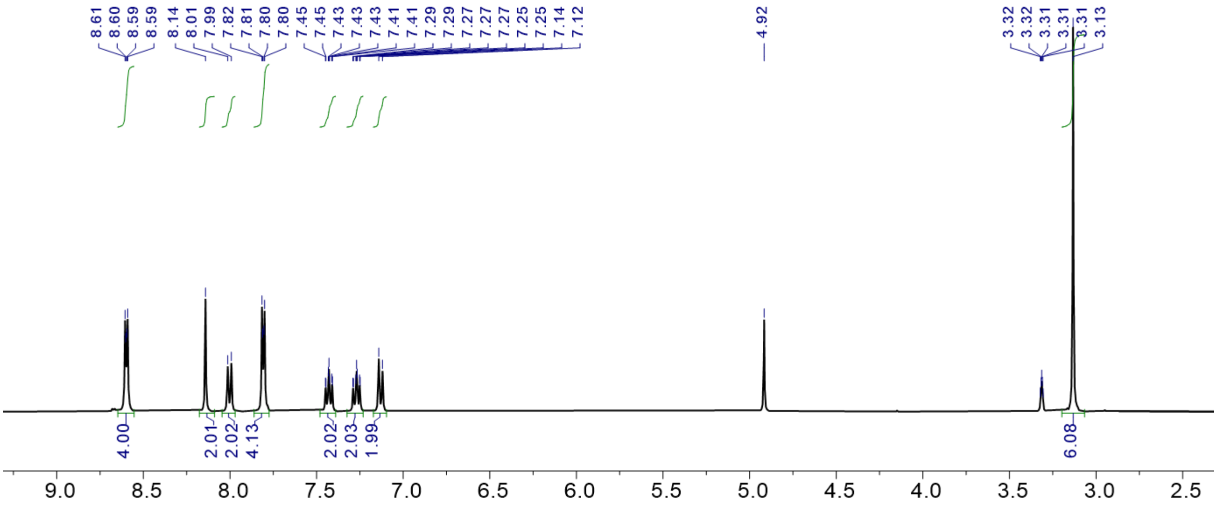

Figure S2. ^{1}H NMR spectrum (CD_3OD, 298 K, 400 MHz) of compound *R*-**B**.

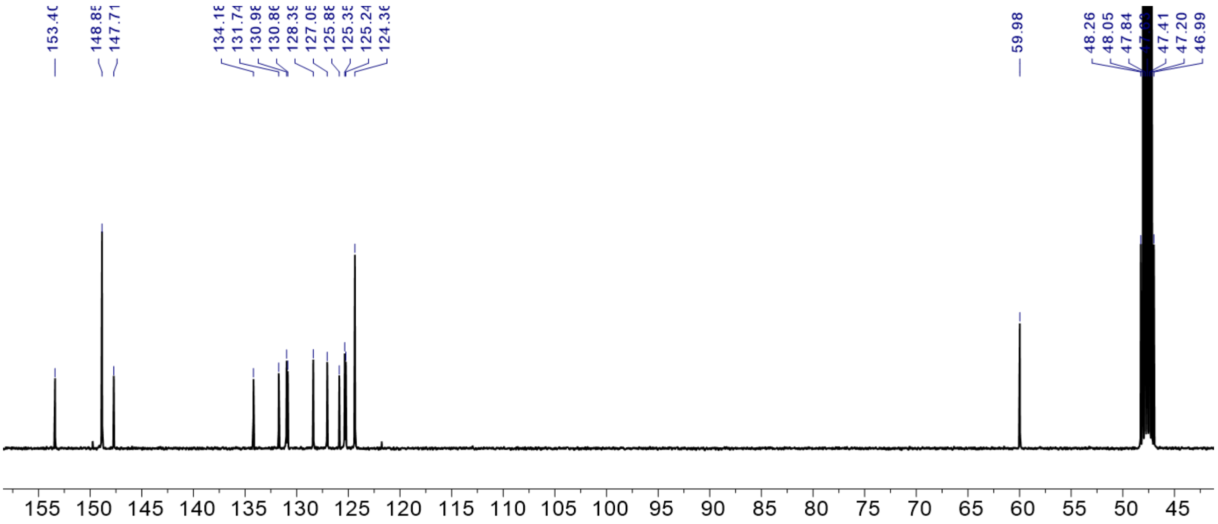

Figure S3. ^{13}C NMR spectrum (CD_3OD, 298 K, 100 MHz) of compound *R*-**B**.

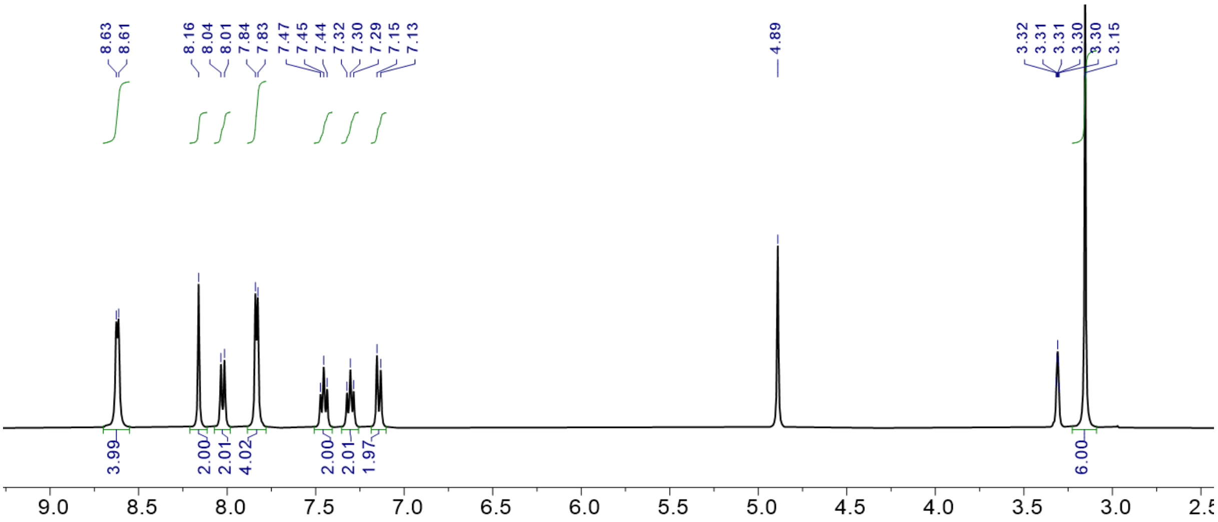

Figure S4. ^{1}H NMR spectrum (CD_3OD, 298 K, 400 MHz) of compound *S*-**B**.

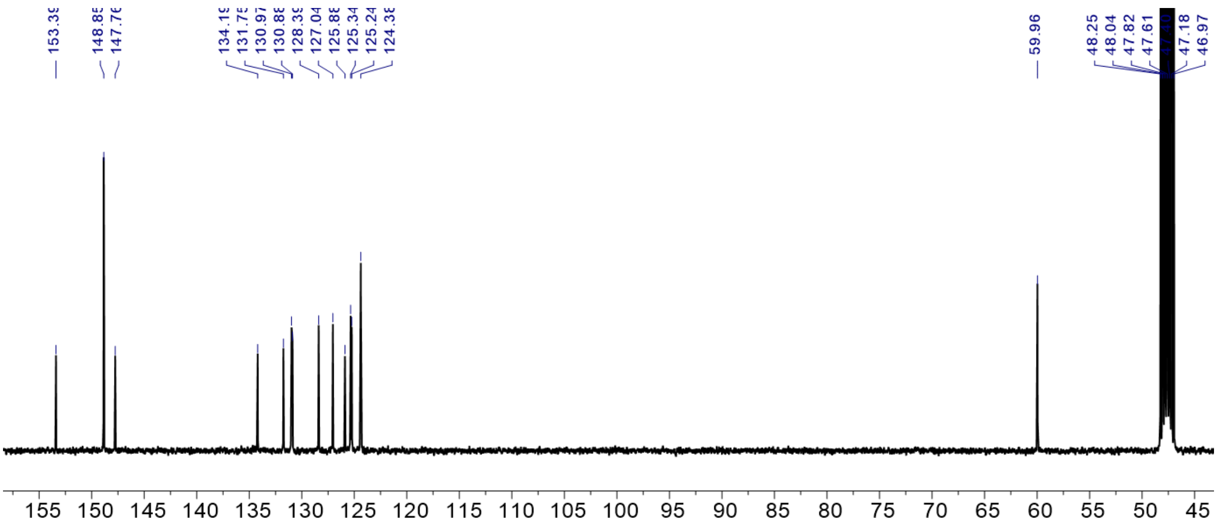

Figure S5. ^{13}C NMR spectrum (CD_3OD, 298 K, 100 MHz) of compound *S*-**B**.

Compound C was synthesized according to the reported procedures [1].

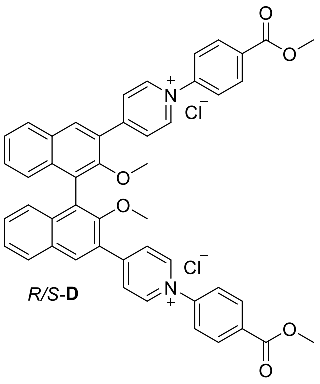

R/*S*-**D**

R/*S*-**D:** In a 250 mL round-bottom flask, *R*/*S*-**D** (410 mg; 0.875 mmol), compound **C** (1.60 g; 3.42 mmol), and $Cu(OAc)_2{\cdot}H_2O$ (52.0 mg; 0.260 mmol) were dissolved in DMF (10 mL). The mixture was heated at 100 °C for 24 hours under nitrogen atmosphere. After cooling to room temperature, the mixture was poured into diethyl ether, and a significant amount of precipitate formed immediately, which was isolated through centrifugation. The yellow solid was then dissolved in acetone (15 mL), and TBACl was added to the solution, resulting in a large amount of yellow precipitate. After filtration and washing with diethyl ether (3 × 12 mL), the precipitate was collected and was dried under vacuum to give *R*/*S*-**D** as a yellow solid (500 mg, 70.6% yield).

^{1}**H NMR** (*R*-**D**) (CD_3OD, 298 K, 300 MHz): δ 9.36 (d, *J* = 6.9 Hz, 4H), 8.77 (d, *J* = 6.9 Hz, 4H), 8.66 (s, 2H), 8.40 (d, *J* = 8.7 Hz, 4H), 8.23 (d, *J* = 8.2 Hz, 2H), 8.07 (d, *J* = 8.7 Hz, 4H), 7.62 (t, *J* = 7.0 Hz, 2H), 7.56 – 7.46 (m, 2H), 7.27 (d, *J* = 8.5 Hz, 2H), 4.00 (s, 6H), 3.37 (s, 6H) ppm. ^{13}**C NMR** (*R*-**D**) (CD_3OD, 298 K, 100 MHz): δ 165.6, 156.8, 153.3, 145.7, 144.1, 135.5, 133.8, 132.9, 131.4, 130.8, 129.4, 129.1, 128.2, 127.6, 126.3, 126.0, 125.3, 124.6, 61.0, 51.9 ppm.

^{1}**H NMR** (*S*-**D**) (CD_3OD, 298 K, 300 MHz): δ 9.35 (d, *J* = 6.9 Hz, 4H), 8.76 (d, *J* = 7.0 Hz, 4H), 8.65 (s, 2H), 8.40 (d, *J* = 8.7 Hz, 4H), 8.22 (d, *J* = 8.2 Hz, 2H), 8.06 (d, *J* = 8.7 Hz, 4H), 7.62 (ddd, *J* = 8.1, 6.9, 1.1 Hz, 2H), 7.51 (ddd, *J* = 8.3, 6.8, 1.3 Hz, 2H), 7.27 (d, *J* = 8.5 Hz, 2H), 4.00 (s, 6H), 3.37 (s, 6H) ppm. ^{13}**C NMR** (*S*-**D**) (CD_3OD, 298 K, 100 MHz): δ 165.6, 156.8, 153.3, 145.7, 144.1, 135.5, 133.8, 132.9, 131.4, 130.8, 129.4, 129.1, 128.2, 127.6, 126.3, 126.0, 125.3, 124.6, 61.0, 51.9 ppm.

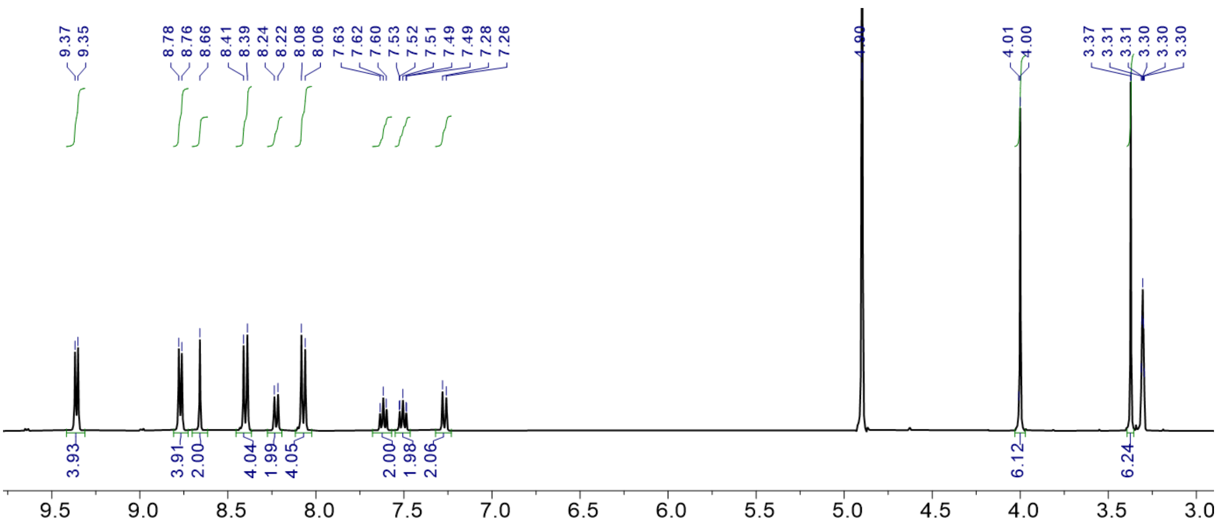

Figure S6. ^{1}H NMR spectrum (CD_3OD, 298 K, 400 MHz) of *R*-**D**.

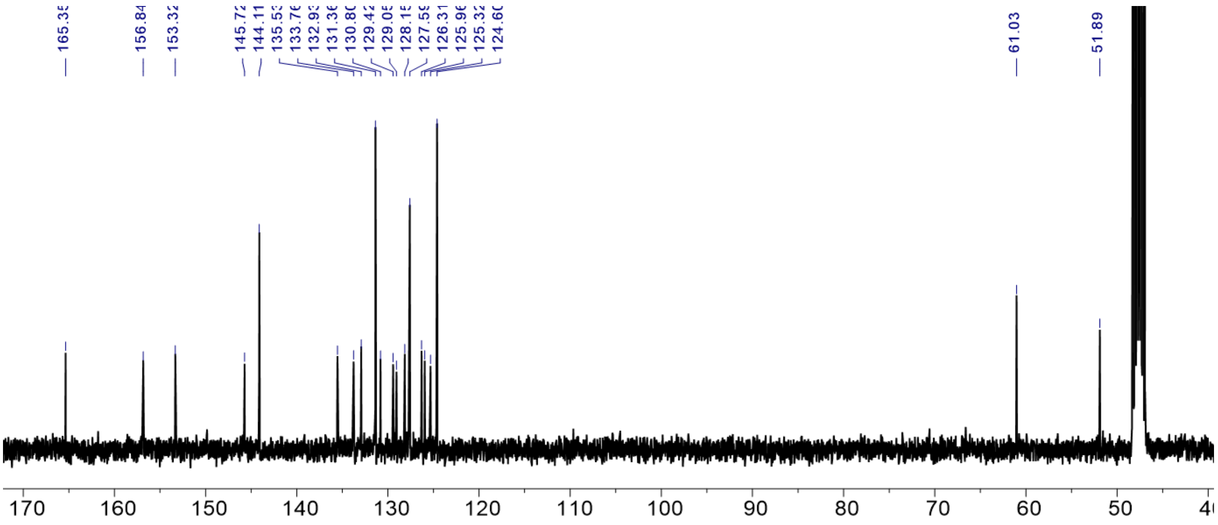

Figure S7. ^{13}C NMR spectrum (CD_3OD, 298 K, 100 MHz) of *R*-**D**.

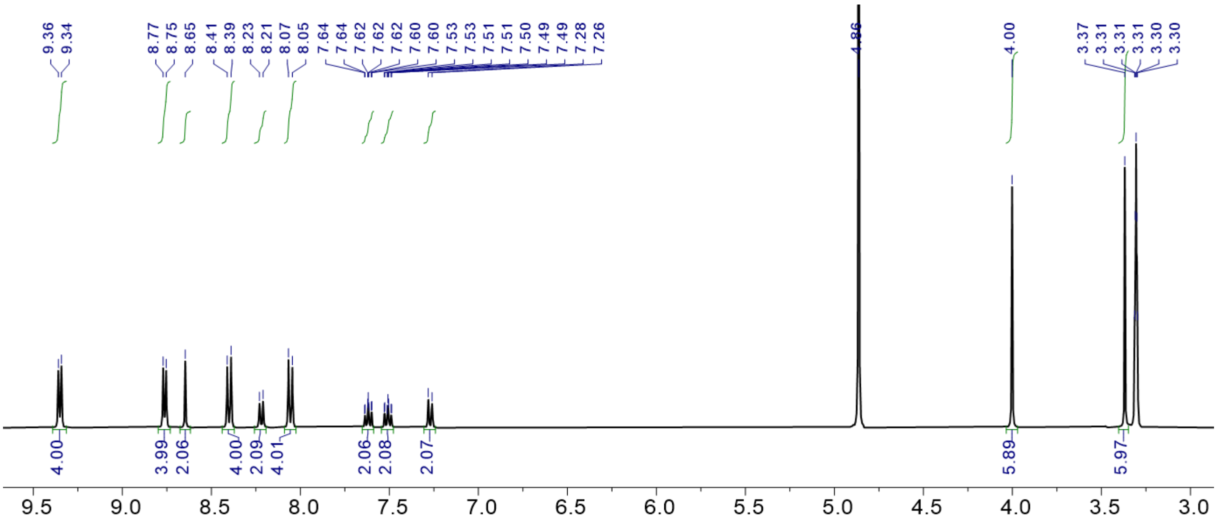

Figure S8. ^{1}H NMR spectrum (CD_3OD, 298 K, 400 MHz) of *S*-**D**.

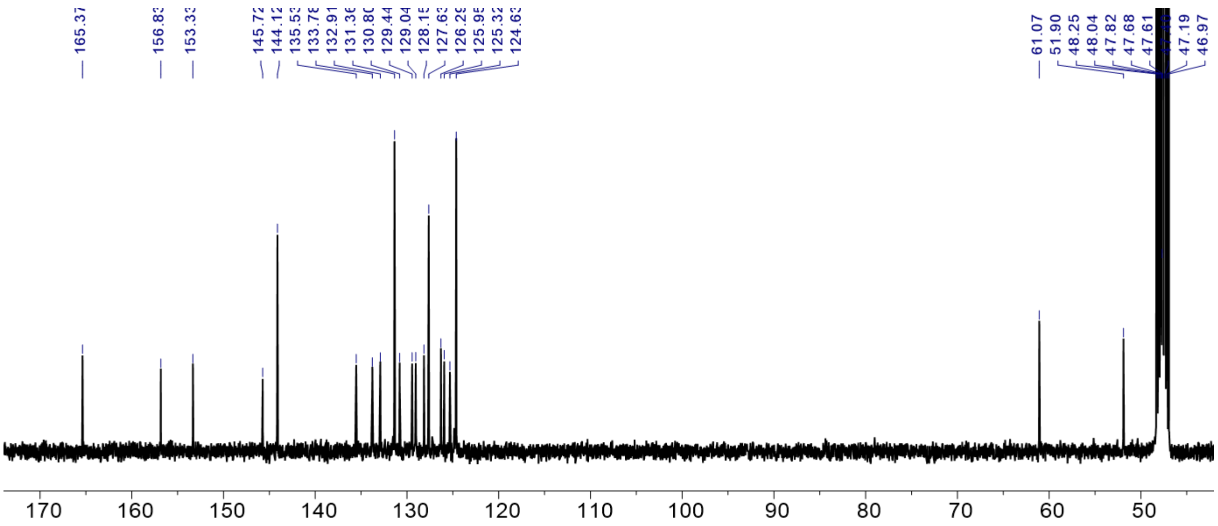

Figure S9. ^{13}C NMR spectrum (CD_3OD, 298 K, 100 MHz) of *S*-**D**.

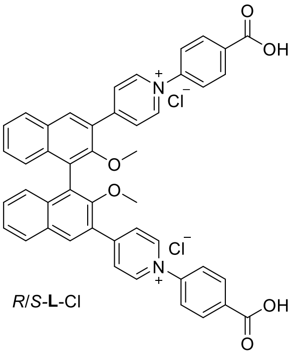

R/*S*-**L**-Cl

R/*S*-**L**-Cl: In a 250 mL round-bottom flask, *R*/*S*-**D** (120 mg, 0.148 mmol) was dissolved in 60 mL of water. A solution of HCl (36%, 60 mL) was then added to the flask. The reaction mixture was gradually heated to 100 °C and maintained overnight. After cooling to room temperature, the crude product was precipitated and was obtained through filtration. The crude material was further washed with 5 mL of acetone, giving pure *R*/*S*-**L**-Cl as a yellow solid (90 mg, 78% yield).

^{1}H NMR (*R*-**L**-Cl) (CD_3OD, 298 K, 400 MHz): δ 9.34 (d, J = 7.0 Hz, 4H), 8.74 (d, J = 7.0 Hz, 4H), 8.64 (s, 2H), 8.34 (d, J = 8.7 Hz, 4H), 8.22 (d, J = 8.2 Hz, 2H), 7.96 (d, J = 8.7 Hz, 4H), 7.62 (ddd, J = 8.1, 6.8, 1.2 Hz, 2H), 7.51 (ddd, J = 8.3, 6.9, 1.3 Hz, 2H), 7.27 (d, J = 8.5 Hz, 2H), 3.37 (s, 6H) ppm. **^{13}C NMR** (*R*-**L**-Cl) (CD_3OD, 298 K, 100 MHz): δ 166.4, 156.8, 153.3, 145.6, 144.1, 135.5, 133.8, 133.7, 131.6, 130.8, 129.4, 129.0, 128.2, 127.6, 126.3, 126.0, 125.3, 124.5, 61.1 ppm.

^{1}H NMR (*S*-**L**-Cl) (CD_3OD, 298 K, 300 MHz): δ 9.36 (d, J = 7.0 Hz, 2H), 8.76 (d, J = 7.0 Hz, 2H), 8.65 (s, 1H), 8.40 (d, J = 8.7 Hz, 1H), 8.22 (d, J = 8.1 Hz, 1H), 8.04 (d, J = 8.7 Hz, 2H), 7.68 – 7.56 (m, 1H), 7.51 (dd, J = 8.4, 7.0 Hz, 1H), 7.27 (d, J = 8.4 Hz, 1H), 3.37 (s, 3H) ppm. **^{13}C NMR** (*S*-**L**-Cl) (CD_3OD, 298 K, 100 MHz): δ 166.4, 156.8, 153.3, 145.6, 144.2, 135.5, 133.8, 133.7, 131.6, 130.8, 129.5, 129.0, 128.2, 127.7, 126.3, 126.0, 125.3, 124.5, 61.2 ppm.

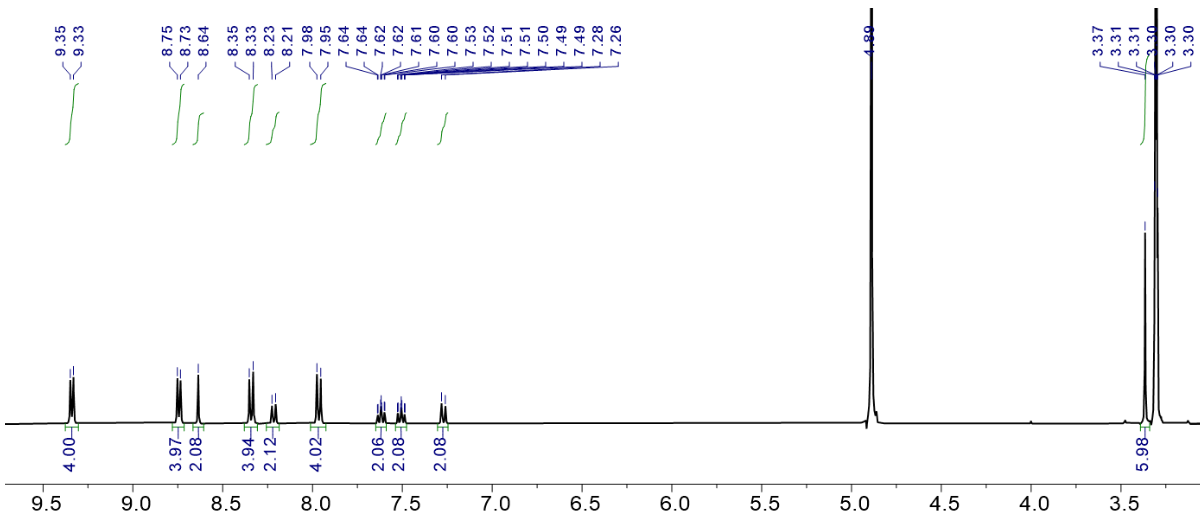

Figure S10. ^{1}H NMR spectrum (CD_3OD, 298 K, 400 MHz) of *R*-**L**-Cl.

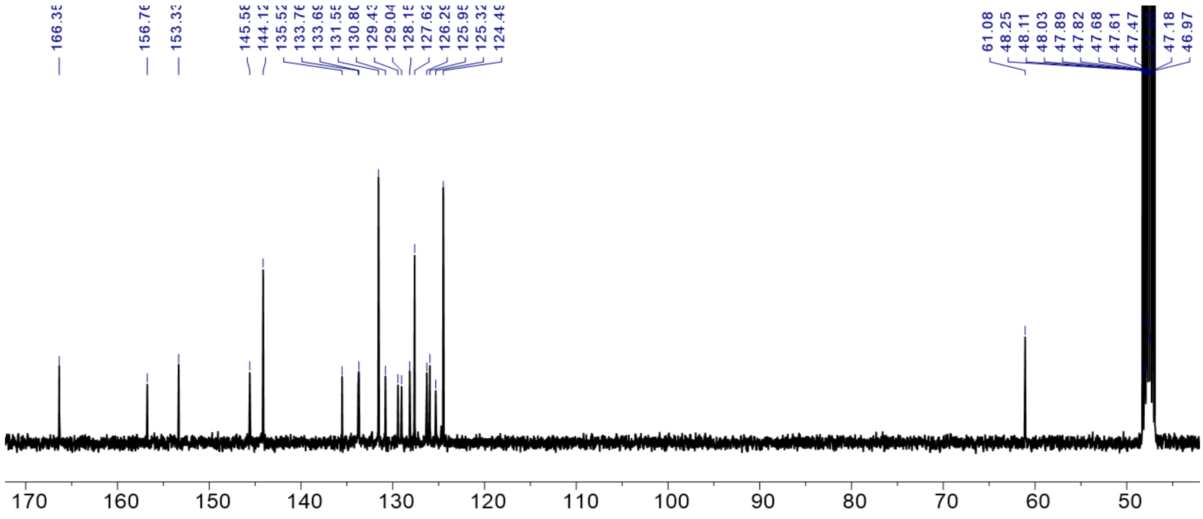

Figure S11. ^{13}C NMR spectrum (CD_3OD, 298 K, 100 MHz) of *R*-**L**-Cl.

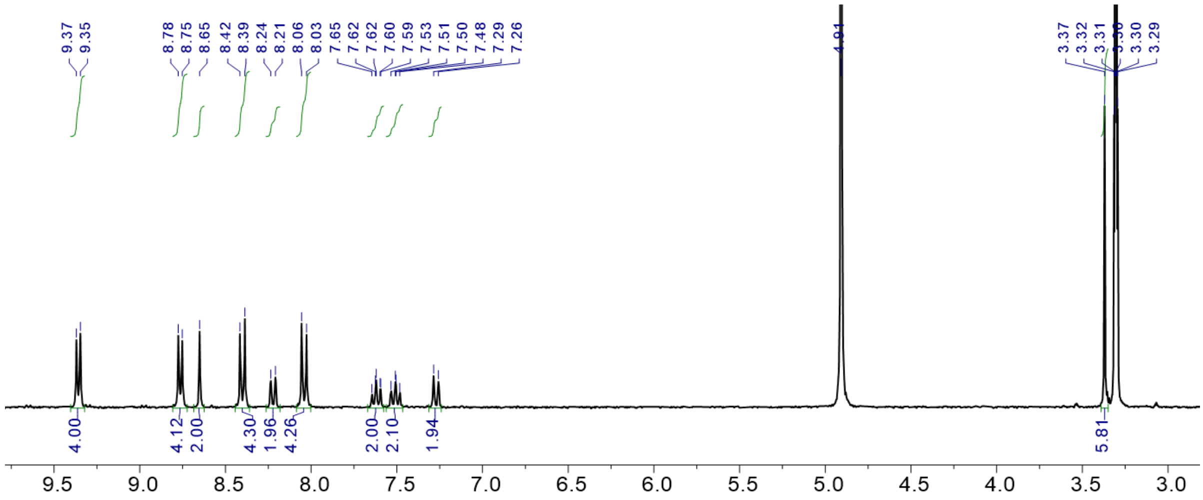

Figure S12. ^{1}H NMR spectrum (CD_3OD, 298 K, 400 MHz) of *S*-**L**-Cl.

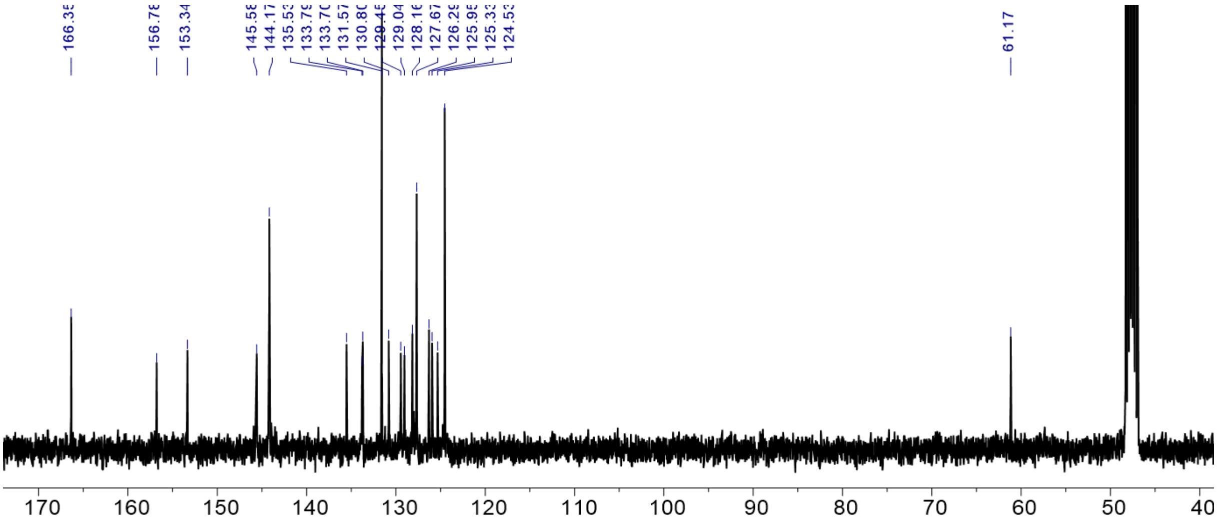

Figure S13. ^{13}C NMR spectrum (CD_3OD, 298 K, 100 MHz) of *S*-**L**-Cl.

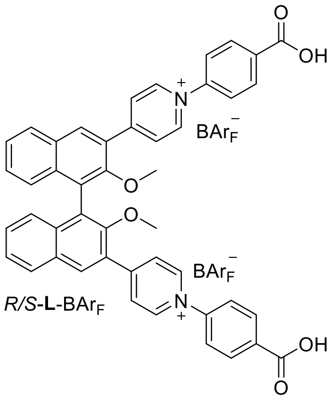

R/*S*-**L**-BAr$_F$ was synthesized via an anion exchange of *R*/*S*-**L**-Cl with NaBAr$_F$. *R*/*S*-**L**-Cl (90 mg, 0.115 mmol) was dissolved in 100 mL of water and slowly heated to 70 °C to ensure complete dissolution. Subsequently, NaBAr$_F$ (204 mg, 0.23 mmol) was dissolved in 1 mL of ethanol and slowly added to the solution of *R*/*S*-**L**-Cl, resulting in the formation of a significant amount of white precipitate. After filtration, the precipitate was washed with water (3 × 15 mL), collected, and dried under vacuum to give a yellow solid (200 mg, 71.4% yield).

^{1}H NMR (*R*-**L**-BAr$_F$) (CD_3OD, 298 K, 400 MHz): *δ* 9.35 (d, *J* = 7.0 Hz, 4H), 8.76 (d, *J* = 6.8 Hz, 4H), 8.64 (s, 2H), 8.40 (d, *J* = 8.4 Hz, 4H), 8.21 (d, *J* = 8.2 Hz, 2H), 8.03 (d, *J* = 7.9 Hz, 4H), 7.68 – 7.55 (m, 26H), 7.54 – 7.46 (m, 2H), 7.27 (d, *J* = 8.4 Hz, 2H), 3.37 (s, 6H) ppm. **^{13}C NMR** (*R*-**L**-BAr$_F$) (CD_3OD, 298 K, 100 MHz): *δ* 166.4, 156.8, 153.3, 145.6, 144.1, 135.5, 133.8, 133.7, 131.6, 130.8, 129.4, 129.0, 128.2, 127.6, 126.3, 126.0, 125.3, 124.5, 61.1 ppm. **^{19}F NMR** (*R*-**L**-BAr$_F$) (CD_3OD, 298 K, 376 MHz): *δ* -64.2 ppm.

^{1}H NMR (*S*-**L**-BAr$_F$) (CD_3OD, 298 K, 300 MHz): δ 9.35 (d, *J* = 7.0 Hz, 4H), 8.76 (d, *J* = 7.0 Hz, 4H), 8.64 (s, 2H), 8.41 (d, *J* = 8.7 Hz, 4H), 8.21 (d, *J* = 8.2 Hz, 2H), 8.03 (d, *J* = 8.7 Hz, 4H), 7.66 – 7.56 (m, 26H), 7.50 (ddd, *J* = 8.2, 6.8, 1.3 Hz, 2H), 7.27 (d, *J* = 8.4 Hz, 2H), 3.37 (s, 6H) ppm. **^{13}C NMR** (*S*-**L**-BAr$_F$) (CD_3OD, 298 K, 100 MHz): *δ* 166.4, 162.2, 161.7, 161.2, 160.8, 157.0, 153.3, 145.5, 144.0, 135.6, 134.4, 133.7, 131.6, 130.8, 129.5, 129.3, 129.3, 129.2(3), 129.2(0), 129.1(7), 129.1(0), 128.9(4), 128.9(1), 128.9(0), 128.8, 128.6, 128.4, 128.1, 127.6, 126.3, 126.0, 125.7, 125.3, 124.4, 123.0, 120.3, 117.2, 117.1, 117.0, 60.9 ppm. **^{19}F NMR** (*S*-**L**-BAr$_F$) (CD_3OD, 298 K, 376 MHz): *δ* -64.3 ppm.

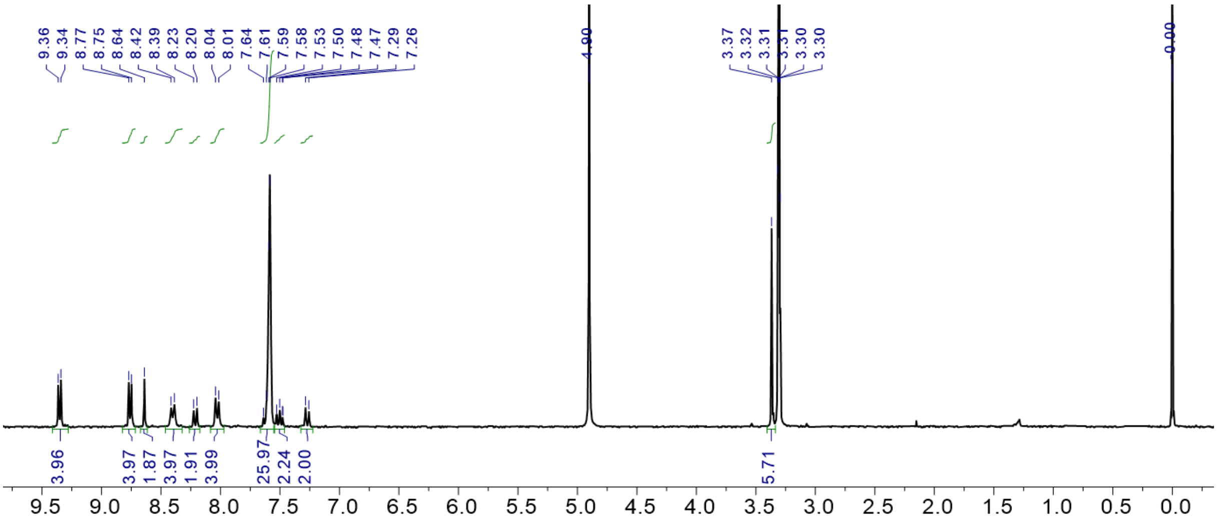

Figure S14. ^{1}H NMR spectrum (CD_3OD, 298 K, 400 MHz) of *R*-**L**-BAr$_F$.

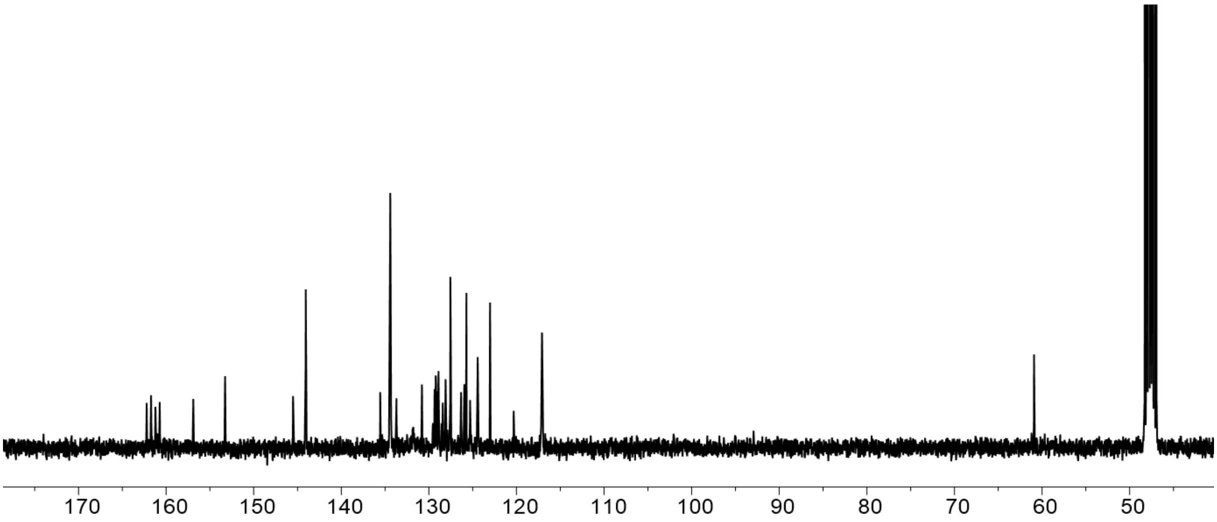

Figure S15. ^{13}C NMR spectrum (CD_3OD, 298 K, 100 MHz) of *R*-**L**-BAr$_F$.

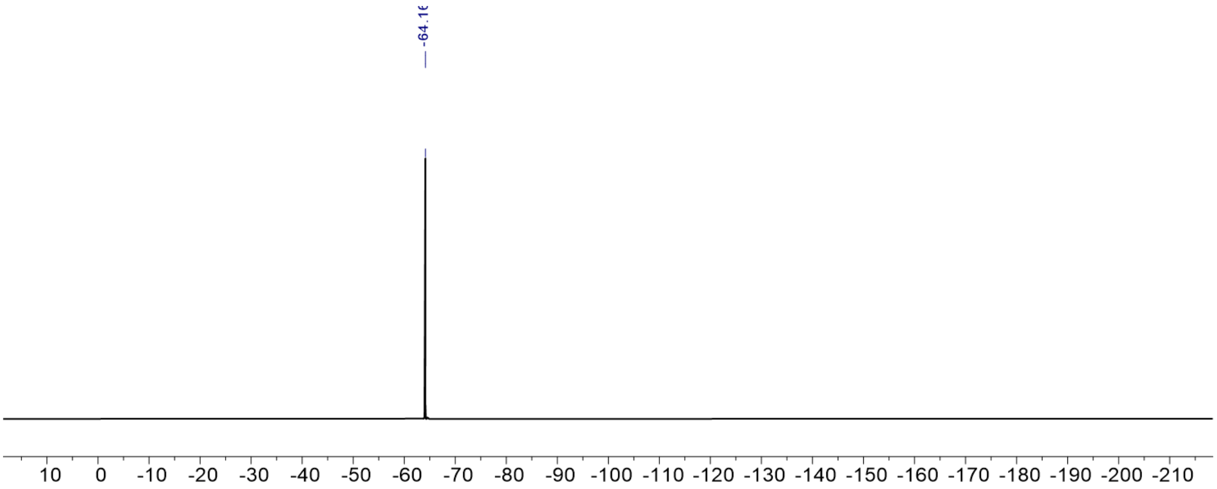

Figure S16. ^{19}F NMR spectrum (CD_3OD, 298 K, 376 MHz) of *R*-**L**-BAr$_F$.

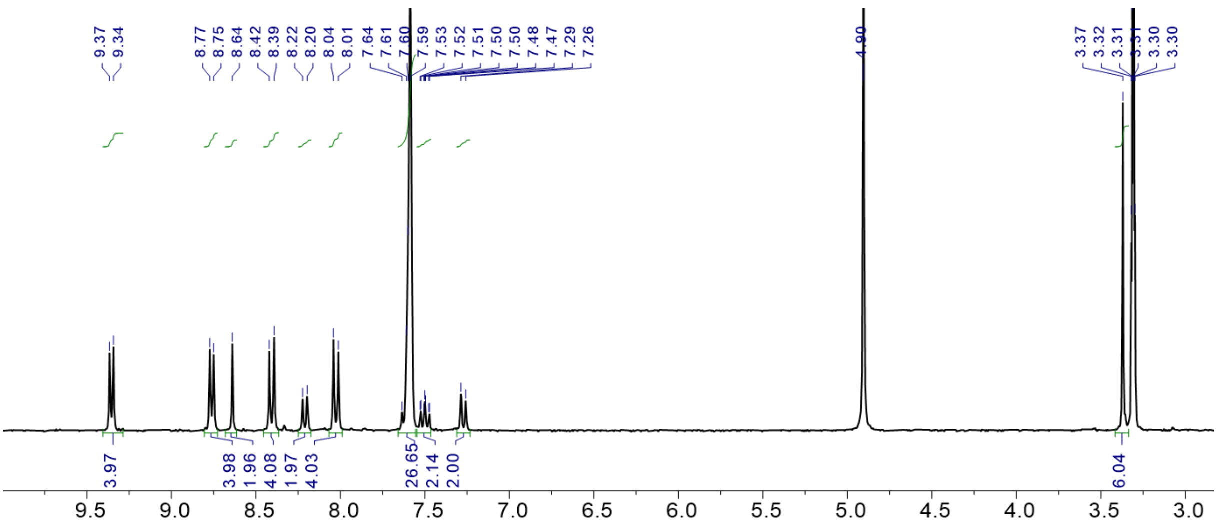

Figure S17. ^{1}H NMR spectrum (CD_3OD, 298 K, 300 MHz) of *S*-**L**-BAr_F.

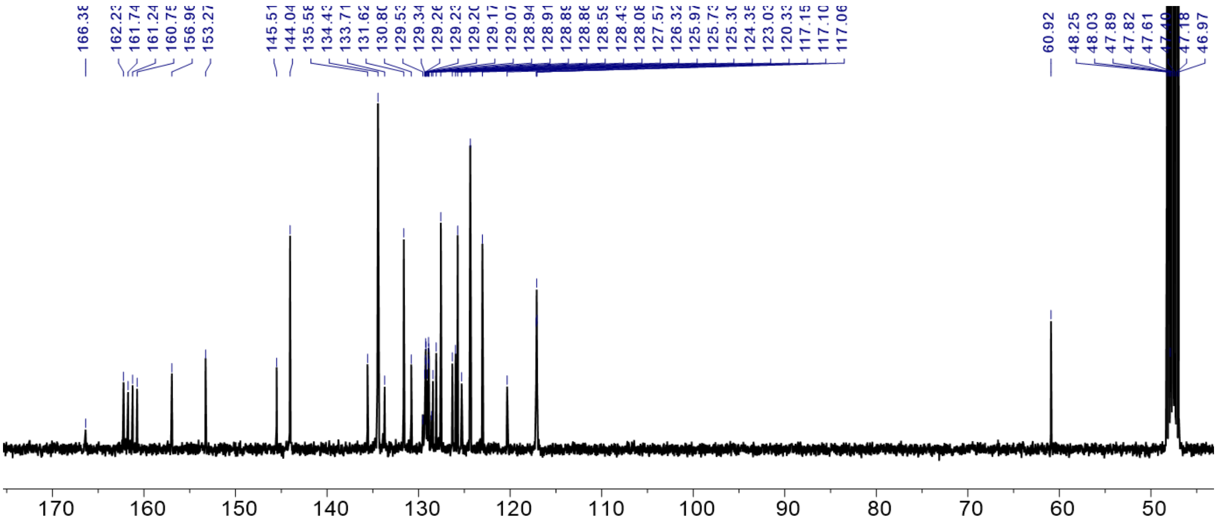

Figure S18. ^{13}C NMR spectrum (CD_3OD, 298 K, 100 MHz) of *S*-**L**-BAr_F.

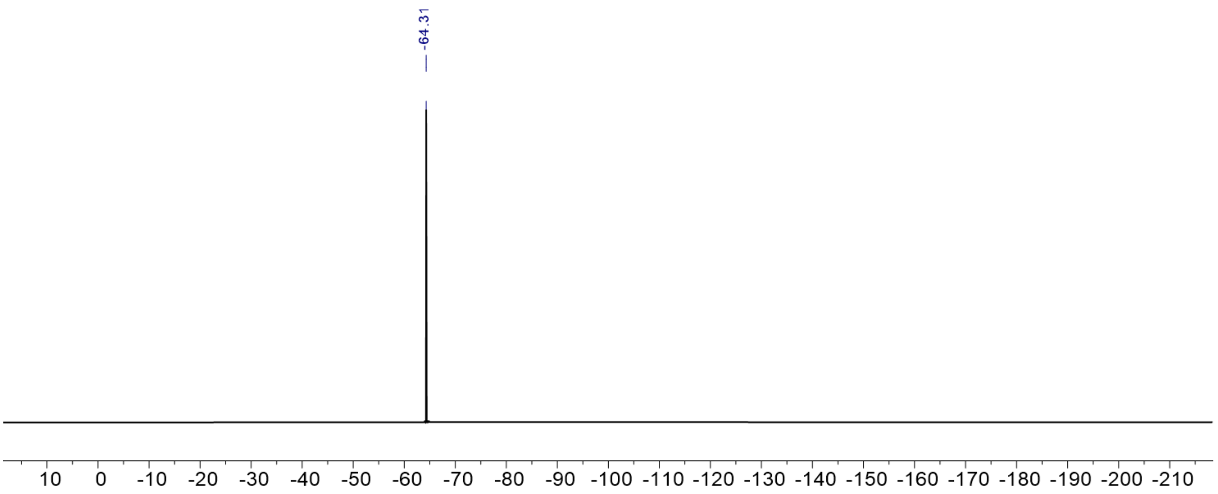

Figure S19. ^{19}F NMR spectrum (CD_3OD, 298 K, 376 MHz) of *S*-**L**-BAr_F.

1.2 Synthesis and characterization of *R*/*S*-1-BAr$_F$

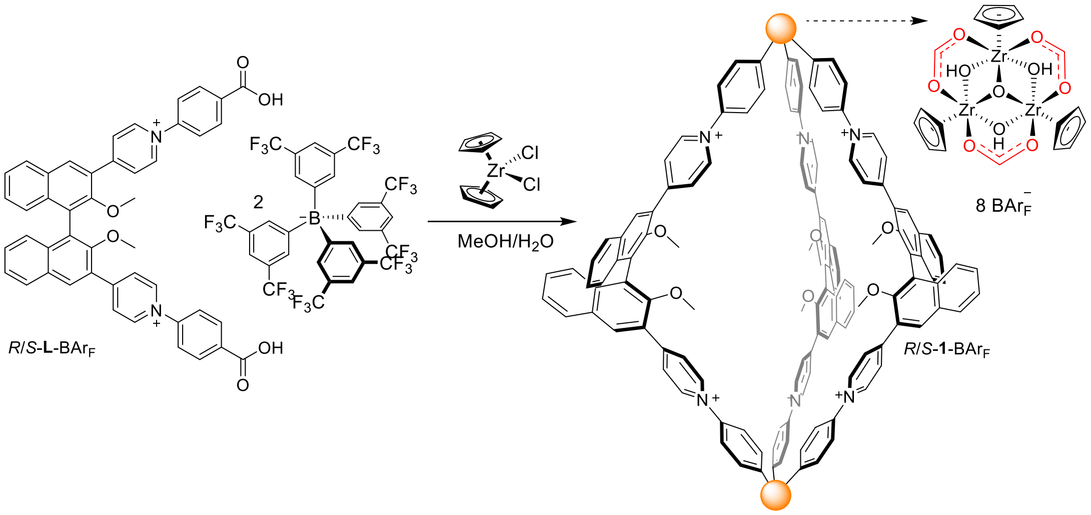

Figure S20. Synthesis of *R*/*S*-**1**-BAr$_F$.

R/*S*-**1**-BAr$_F$**:** *R*/*S*-**L**-BAr$_F$ (20 mg, 8.2 μmol, 1.0 equiv.), Cp_2ZrCl_2 (5.1 mg, 17.4 μmol, 2.1 equiv.), and NaBAr$_F$ (7.3 mg, 8.2 μmol, 1.0 equiv.) were dissolved in a mixture of 3 mL of CH_3OH and 120 μL of H_2O. The reaction mixture was stirred at room temperature overnight. After completion, 12 mL of water was added, resulting in the formation of a large amount of white precipitate. The precipitate was collected by centrifugation, thoroughly washed with water (3 × 12 mL), and dried under vacuum to give *R*/*S*-**1**-BAr$_F$ (21 mg, 76% yield).

^{1}H NMR (*R*-**1**-BAr$_F$) (CD_3OD, 298 K, 400 MHz): *δ* 9.19 (d, J = 6.7 Hz, 12H), 8.68 (d, J = 6.8 Hz, 12H), 8.58 (s, 6H), 8.42 – 8.31 (m, 12H), 8.14 (d, J = 8.2 Hz, 6H), 8.00 – 7.88 (m, 12H), 7.64 – 7.51 (m, 102H), 7.44 (dd, J = 8.5, 7.0 Hz, 6H), 7.17 (d, J = 8.7 Hz, 6H), 6.70 (s, 30H), 3.27 (s, 18H) ppm. **^{13}C NMR** (*R*-**1**-BAr$_F$) (CD_3OD, 298 K, 100 MHz): *δ* 171.7, 162.2, 161.7, 161.2, 160.7, 157.1, 153.2, 145.7, 143.8, 135.6, 134.9, 134.4, 133.8, 131.9, 130.7, 129.5, 129.2(3), 129.2(1), 129.2(0), 128.9(2), 128.9(0), 128.8, 128.6, 128.4, 127.7, 127.4, 126.4, 126.0, 125.7, 125.2, 124.5, 123.0, 120.3, 117.1, 116.6, 60.8 ppm. **^{19}F NMR** (*R*-**1**-BAr$_F$) (CD_3OD, 298 K, 376 MHz): *δ* -64.12 ppm.

^{1}H NMR (*S*-**1**-BAr$_F$) (CD_3OD, 298 K, 400 MHz): *δ* 9.19 (d, J = 6.7 Hz, 12H), 8.68 (d, J = 6.6 Hz, 12H), 8.58 (s, 6H), 8.37 (d, J = 8.4 Hz, 12H), 8.14 (d, J = 8.3 Hz, 6H), 7.93 (d, J = 8.4 Hz, 12H), 7.62 – 7.53 (m, 96H), 7.45 (t, J = 7.7 Hz, 6H), 7.17 (d, J = 8.6 Hz, 6H), 6.70 (d, J = 2.4 Hz, 30H), 3.27 (s, 18H) ppm. **^{3}C NMR** (*S*-**1**-BAr$_F$) (CD_3OD, 298 K, 100 MHz): *δ* 171.7, 162.2, 161.7, 161.2, 160.7, 157.1, 153.2, 145.8, 143.8, 135.6, 134.9, 134.4, 133.8, 132.0, 130.7, 129.5, 129.2(1), 129.2(0), 128.9(2), 128.9(0), 128.8, 128.6, 128.4, 127.7, 127.4, 126.4, 126.0, 125.7, 125.2, 124.5, 123.0, 120.3, 117.1, 116.6, 60.8 ppm. **^{19}F NMR** (*S*-**1**-BAr$_F$) (CD_3OD, 298 K, 376 MHz): *δ* -64.12 ppm.

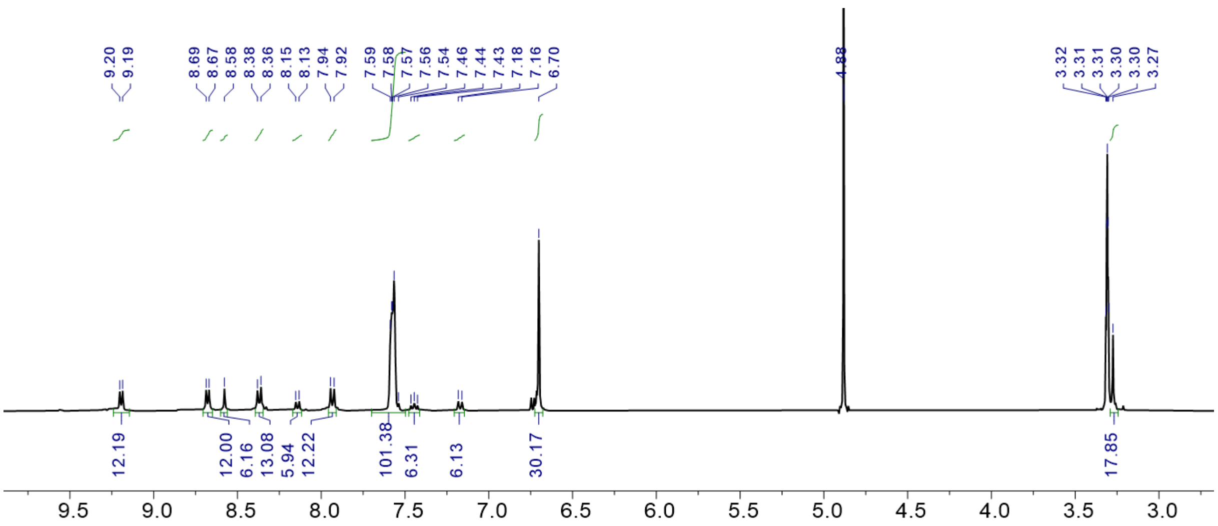

Figure S21. ^{1}H NMR spectrum (CD_3OD, 298 K, 400 MHz) of *R*-**1**-BAr$_F$.

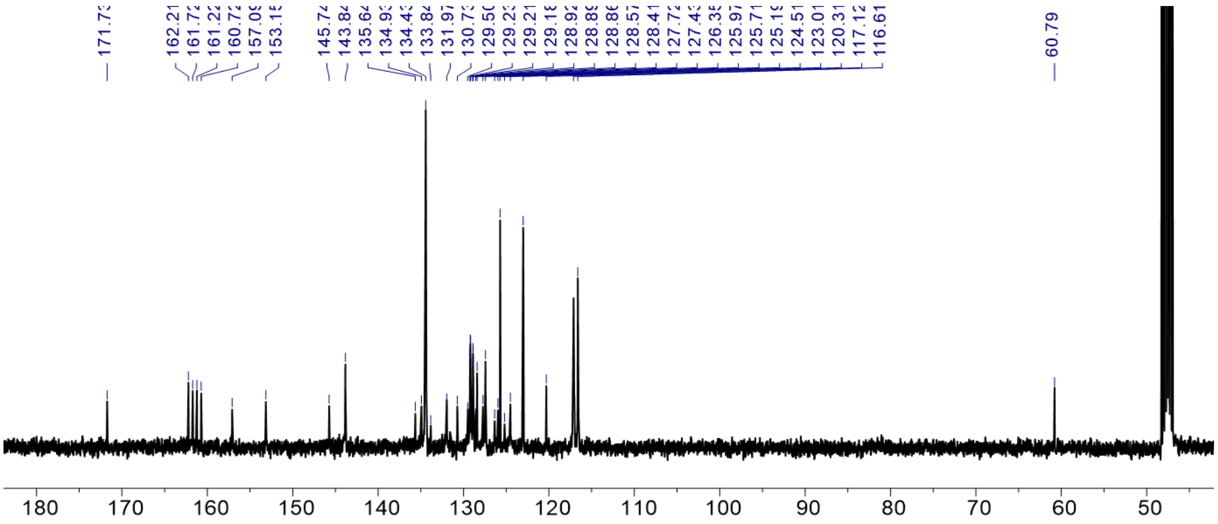

Figure S22. ^{13}C NMR spectrum (CD_3OD, 298 K, 100 MHz) of *R*-**1**-BAr$_F$.

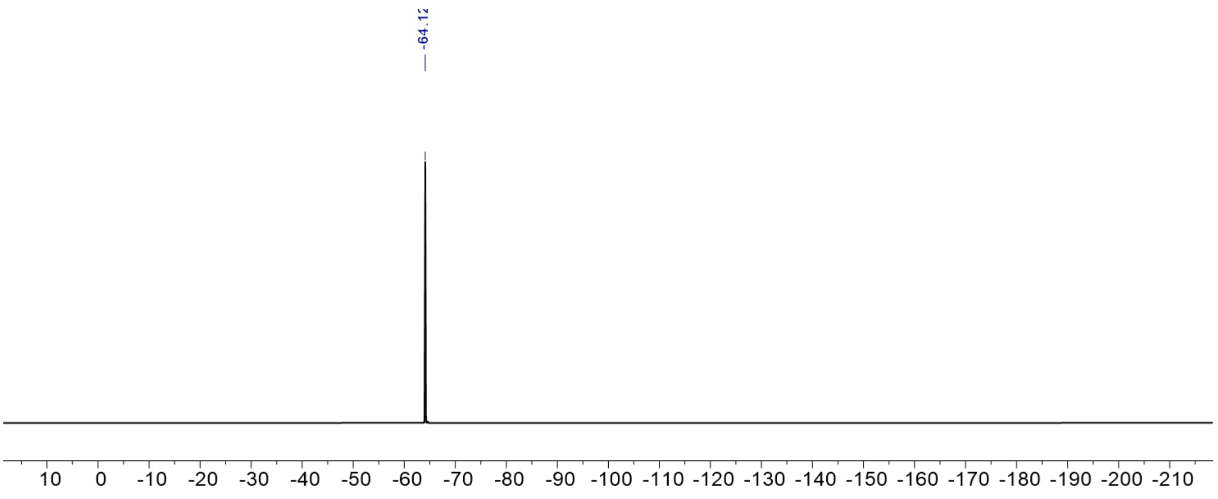

Figure S23. ^{19}F NMR spectrum (CD_3OD, 298 K, 376 MHz) of *R*-**1**-BAr$_F$.

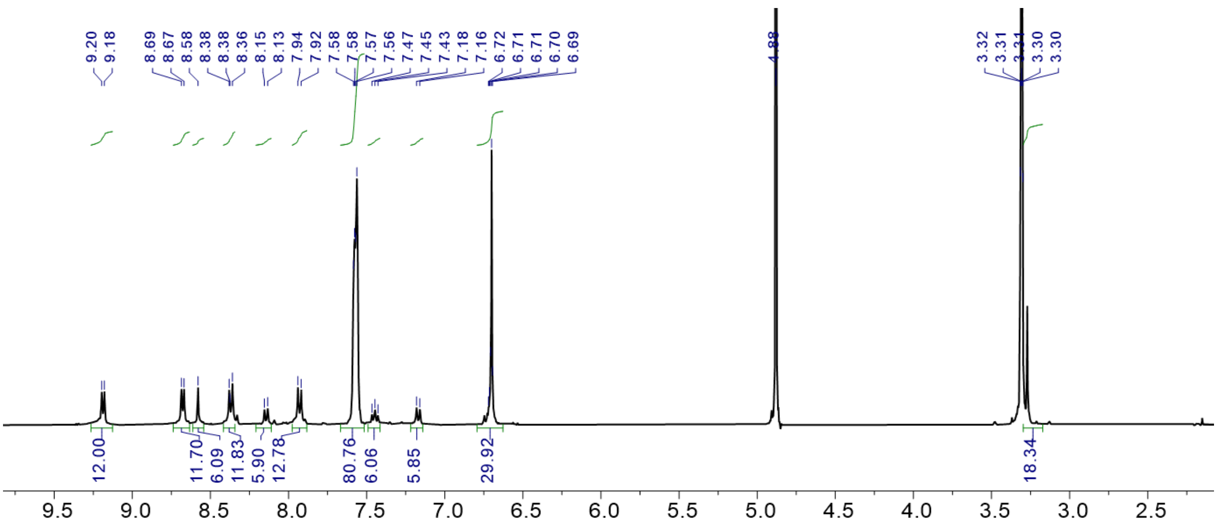

Figure S24. ^{1}H NMR spectrum (CD_3OD, 298 K, 400 MHz) of *S*-**1**-BAr_F.

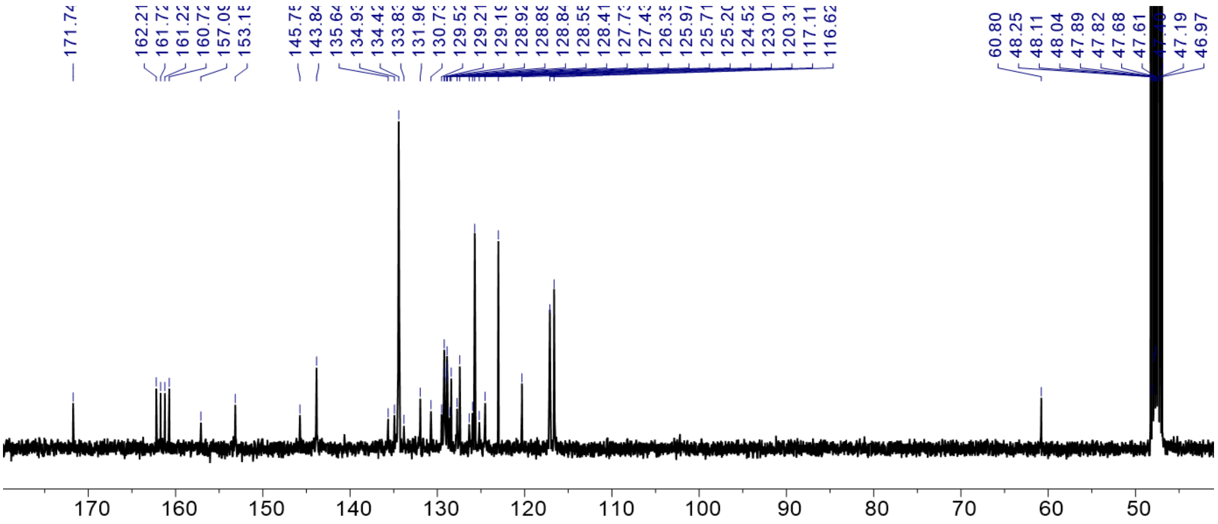

Figure S25. ^{13}C NMR spectrum (CD_3OD, 298 K, 100 MHz) of *S*-**1**-BAr_F.

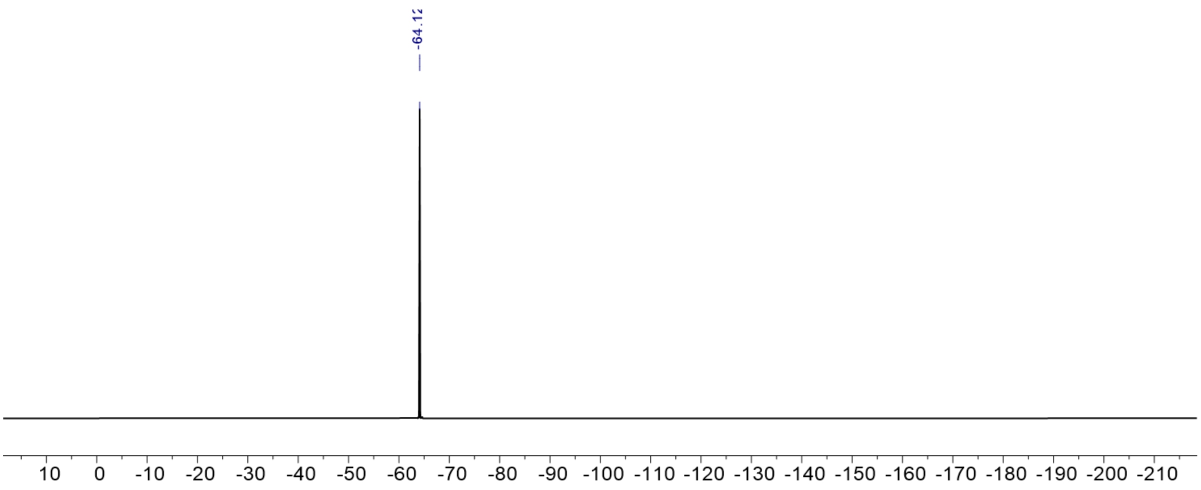

Figure S26. ^{19}F NMR spectrum (CD_3OD, 298 K, 376 MHz) of *S*-**1**-BAr_F.

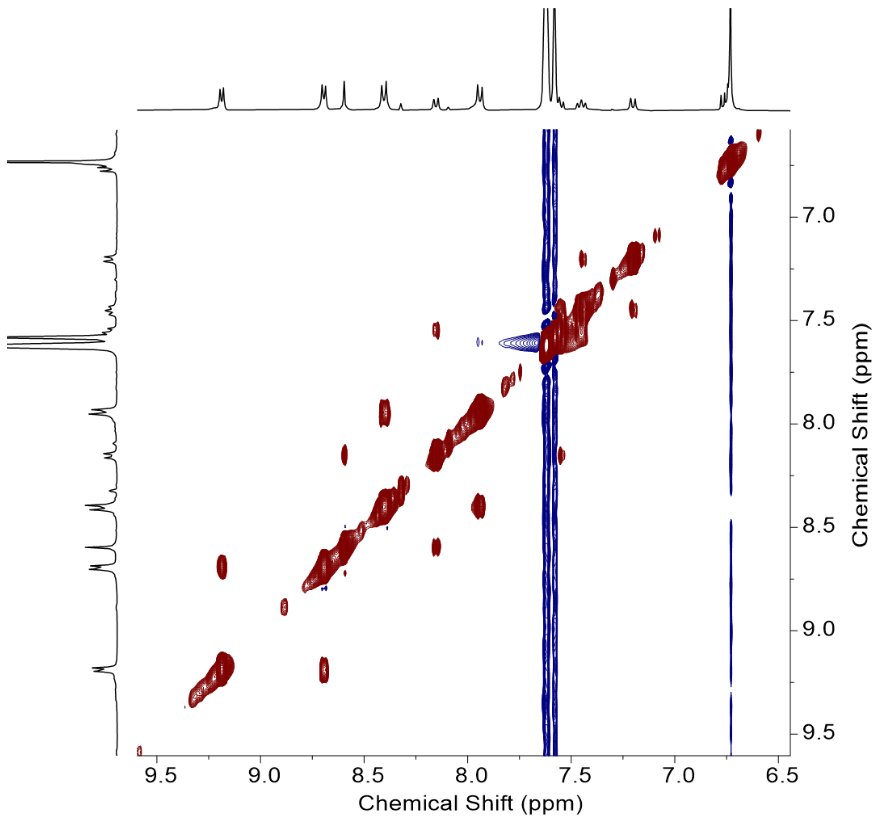

Figure S27. ^1H-^1H COSY spectrum (CD_3OD, 298 K, 500 MHz) of *R*-**1**-BAr$_F$.

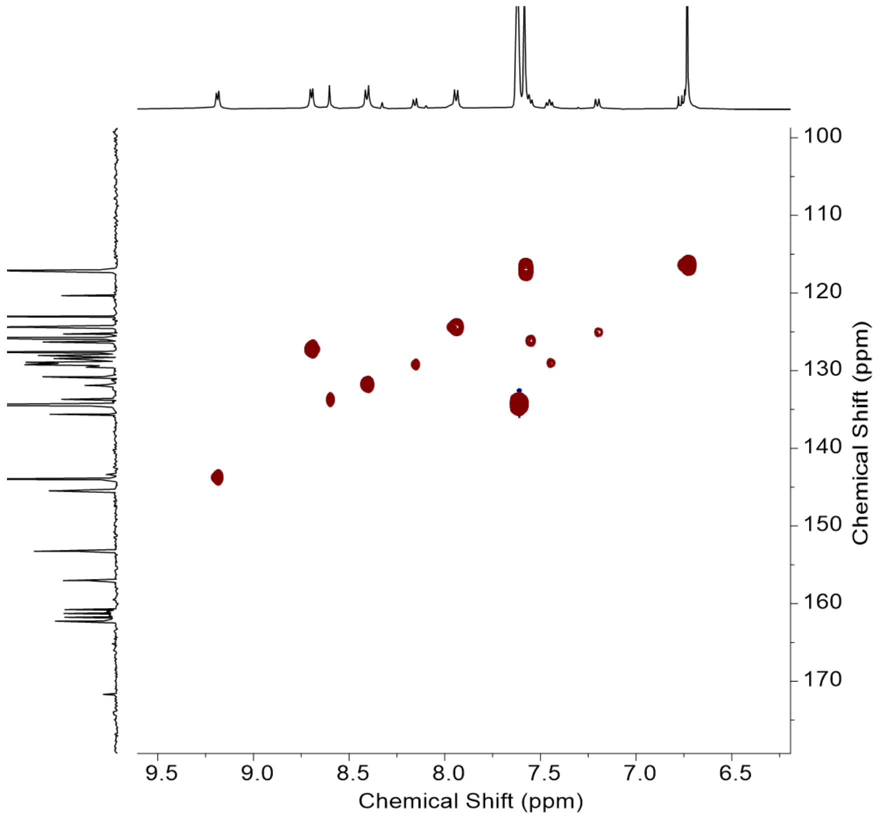

Figure S28. ^1H-^{13}C HSQC spectrum (CD_3OD, 298 K, 500 MHz) of *R*-**1**-BAr$_F$.

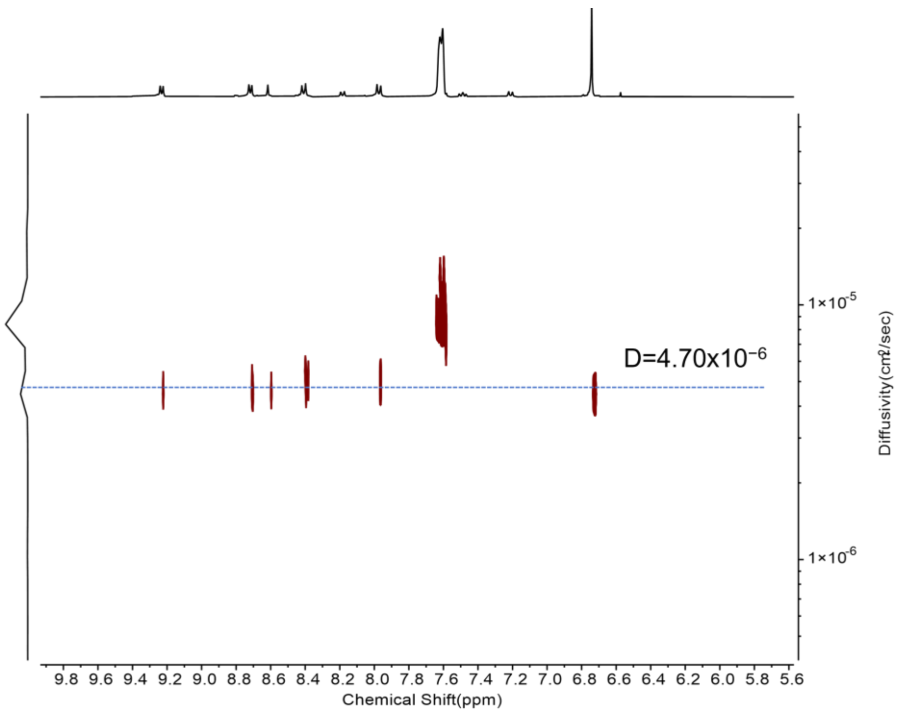

Figure S29. DOSY spectrum (CD_3OD, 298 K, 500 MHz) of *R*-**1**-BAr_F.

Using the Stokes-Einstein equation shown below, the size of the helicate cage in solution was estimated.

$$D = \frac{k_B T}{6\pi\eta r_H}$$

D is the diffusion coefficient of the particle; k_B is the Boltzmann constant (1.38×10^{-23} J/K); *T* is the absolute temperature; *η* is the viscosity of the solvent; *r* is the effective radius of the particle. Calculations show that the effective diameter of the coordination cage is 1.6 nm.

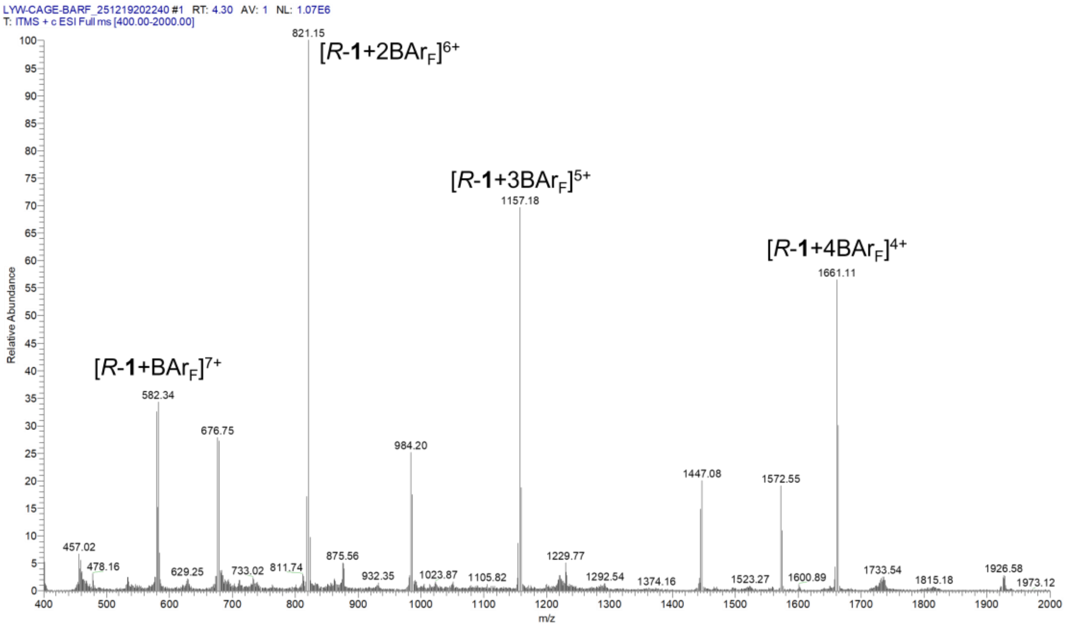

Figure S30. Low-resolution ESI-mass spectrum of *R*-**1**-BAr_F.

1.3 Synthesis and characterization of ***R*/*S*-1-OTf**

Synthesis of *R*/*S*-1-OTf: *R*/*S*-**1**-BAr$_F$ (50 mg, 4.95 µmol, 1.00 equiv.) was dissolved in 2 mL of CH_3OH, followed by the addition of TBAOTf (80 mg, 205 µmol, 41 equiv.). Upon adding13 mL of diethyl ether into the reaction, a significant amount of precipitate formed immediately, which was isolated through centrifugation. The solid thus obtained still contains a minor amount of BAr_F^- in addition to OTf^- as the counteranions. To further minimize the BAr_F^- content, the solid was dissolved in 3 mL of CH_3OH, and diethyl ether (12 mL) was added again to the solution to precipitate **1**-OTf. The precipitate was collected and was dried under vacuum to give **1**-OTf (15 mg, 69% yield).

^{1}H NMR (*R*-**1**-OTf) (CD_3OD, 298 K, 400 MHz) δ 9.16 (d, *J* = 6.6 Hz, 12H), 8.64 (d, *J* = 6.6 Hz, 12H), 8.53 (s, 6H), 8.35 (d, *J* = 8.6 Hz, 12H), 8.12 (d, *J* = 8.2 Hz, 6H), 7.95 (d, *J* = 8.6 Hz, 12H), 7.56 (t, *J* = 7.5 Hz, 6H), 7.46 (t, *J* = 7.8 Hz, 6H), 7.18 (d, *J* = 8.7 Hz, 6H), 6.72 (s, 30H), 3.34 (s, 9H) ppm. **^{19}F NMR** (*R*-**1**-OTf) (CD_3OD, 298 K, 376 MHz): *δ* -79.9 ppm.

^{1}H NMR (*S*-**1**-OTf) (CD_3OD, 298 K, 400 MHz) δ 9.16 (d, *J* = 6.6 Hz, 12H), 8.64 (d, *J* = 6.6 Hz, 12H), 8.53 (s, 6H), 8.35 (d, *J* = 8.6 Hz, 12H), 8.12 (d, *J* = 8.2 Hz, 6H), 7.95 (d, *J* = 8.6 Hz, 12H), 7.56 (t, *J* = 7.5 Hz, 6H), 7.46 (t, *J* = 7.8 Hz, 6H), 7.18 (d, *J* = 8.7 Hz, 6H), 6.72 (s, 30H), 3.34 (s, 9H) ppm. **^{19}F NMR** (CD_3OD, 298 K, 376 MHz): *δ* -79.9 ppm.

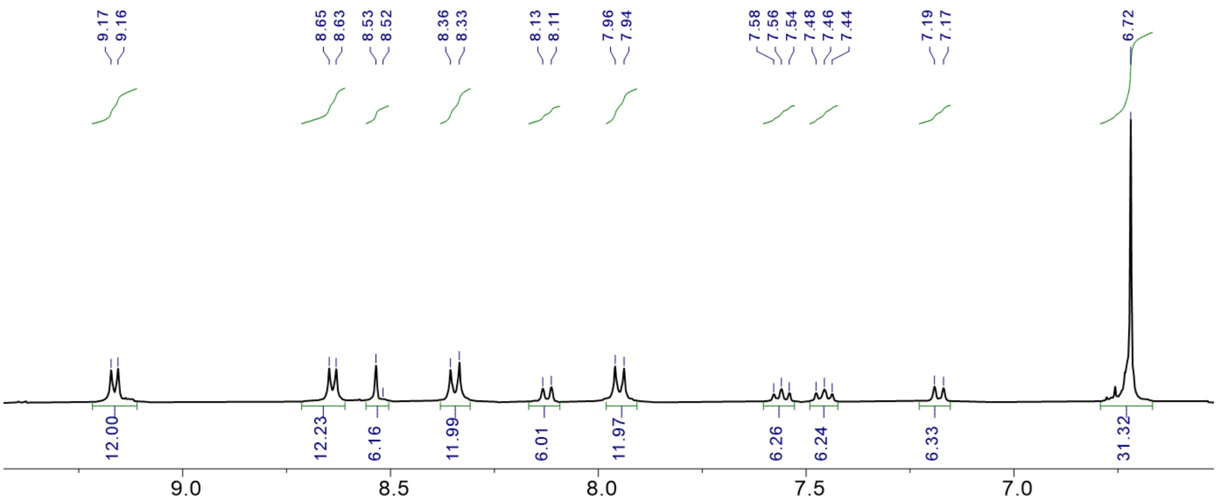

Figure S31. ^{1}H NMR spectrum (CD_3OD, 298 K, 300 MHz) of *R*-**1**-OTf.

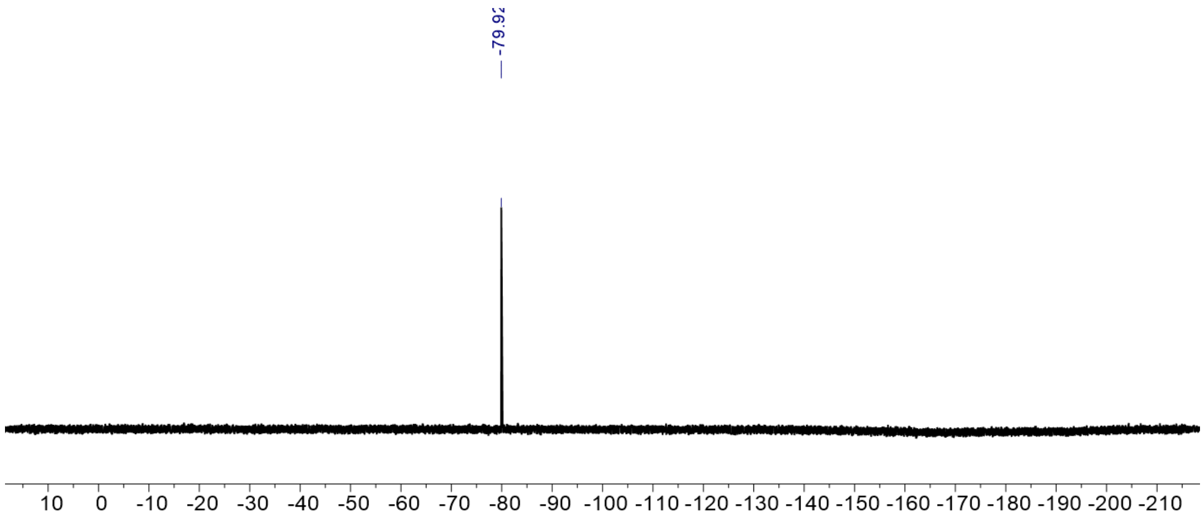

Figure S32. ^{19}F NMR spectrum (CD_3OD, 298 K, 376 MHz) of *R*-**1**-OTf.

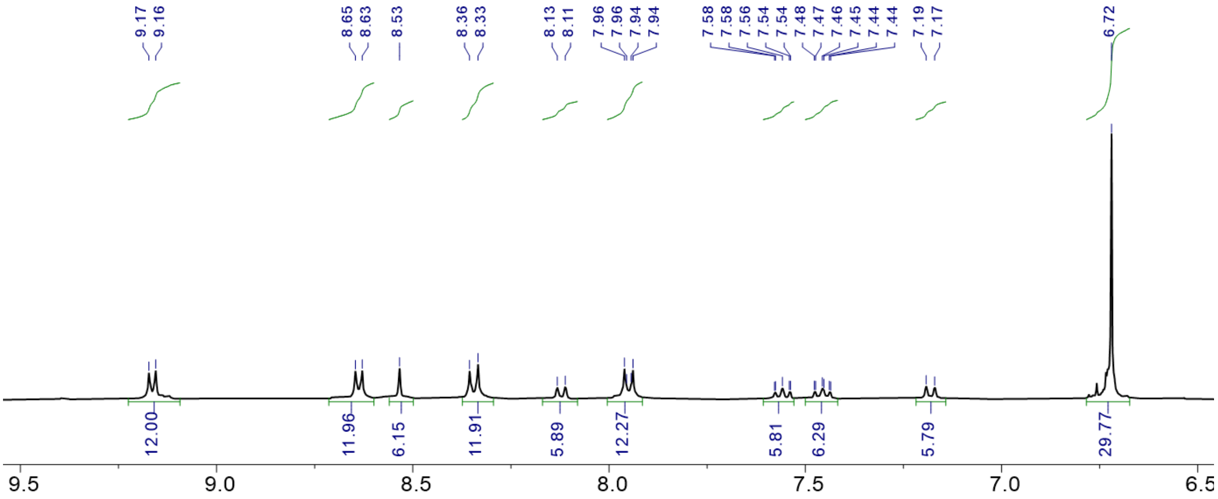

Figure S33. ^{1}H NMR spectrum (CD_3OD, 298 K, 300 MHz) of *R*-**1**-OTf.

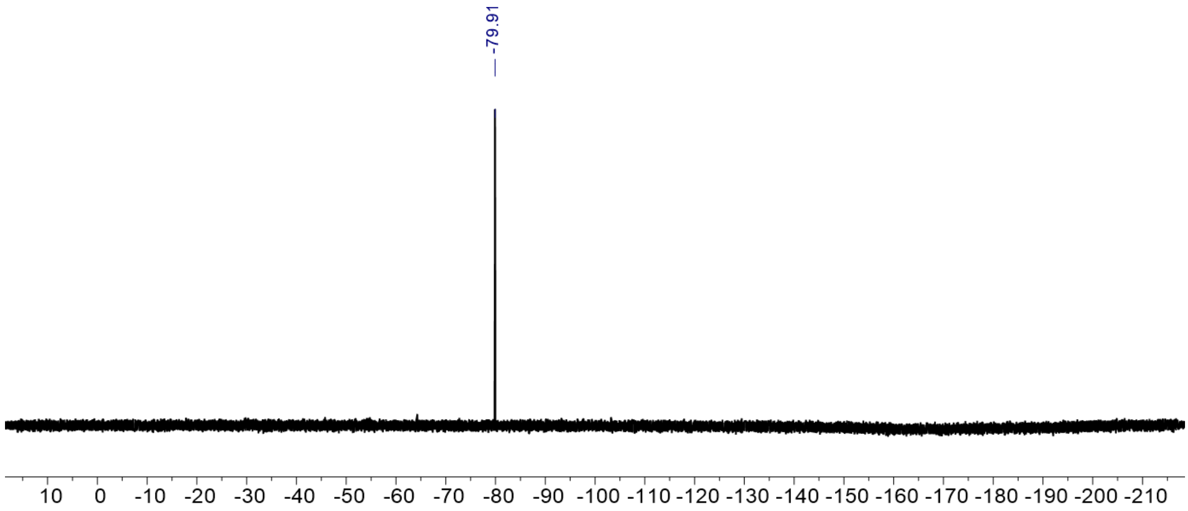

Figure S34. ^{19}F NMR spectrum (CD_3OD, 298 K, 376 MHz) of *R*-**1**-OTf.

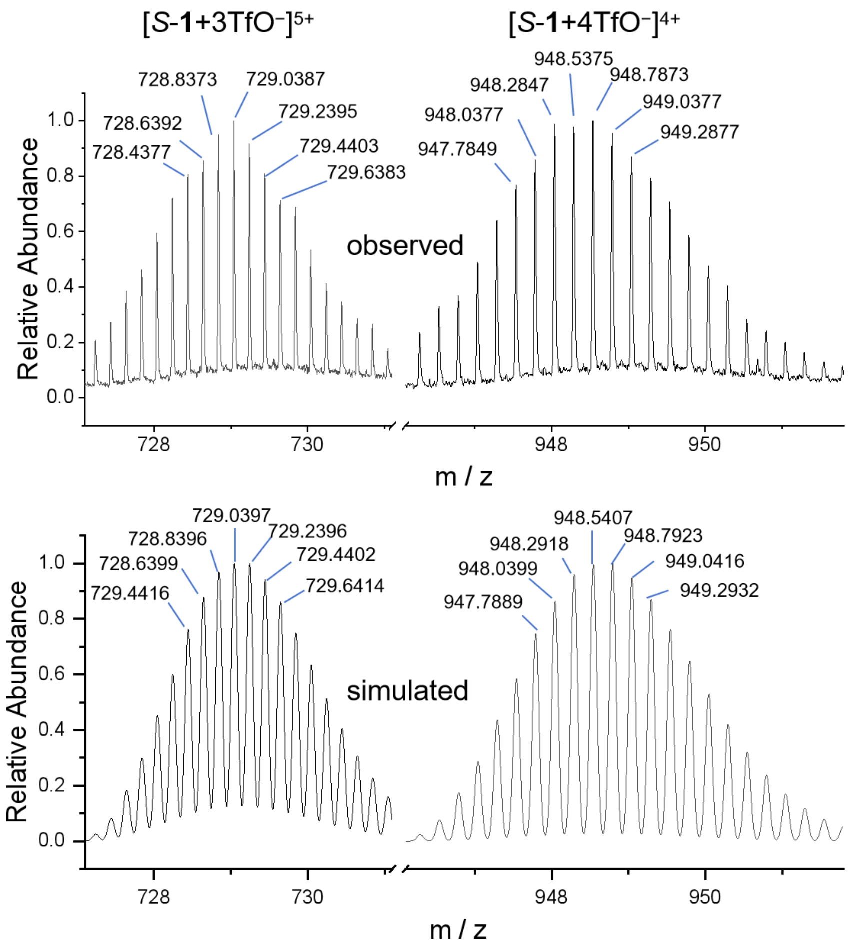

Figure S35. Experimental (top) and simulated (bottom) high-resolution ESI-mass spectra of *S*-**1**-OTf showing the 5+ and 4+ peaks.

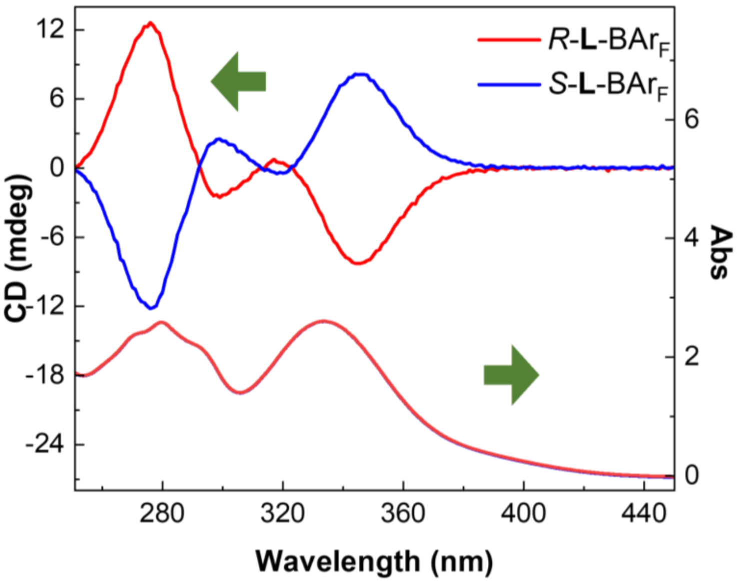

Figure S36. CD and UV-vis spectra of *R*-**L**-BAr_F and *S*-**L**-BAr_F in CH_3OH.

2. Guest binding studies

The anionic guests, including common anions (tetrabutylammonium salts of Cl^-, Br^-, I^-, TfO^-, ClO_4^-, and Tf_2N^-) and *S*-camphorsulfonate (*S*-CS^-, sodium salt), were investigated for the binding properties of *R*/*S*-**1**-BAr_F. ^{1}H NMR titrations were carried out by incrementally adding the CD_3OD solution of the guests into a CD_3OD solution of *R*/*S*-**1**-BAr_F (0.30 mM). The mixture was subjected to ^{1}H NMR. The binding constants were determined using BINDFIT [2] with the data obtained from ^{1}H NMR titrations and are summarized in Table S1.

Table S1. Anionic guests investigated and binding constants with *R*-**1**-BAr_F.

Anion	*S*-CS^-	I^-	Br^-	Cl^-	Tf_2N^-	TfO^-	ClO_4^-
Structure	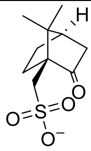	—	—	—	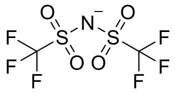	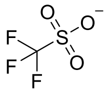	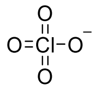
Volume (Å^3)	202[a]	35[b]	28[b]	24[b]	156[b]	85[b]	55[b]
K_a (M^{-1})	1700	690	680	570	230	130	46

[a] Calculated using MoloVol [3]; [b] Obtained from literature [4].

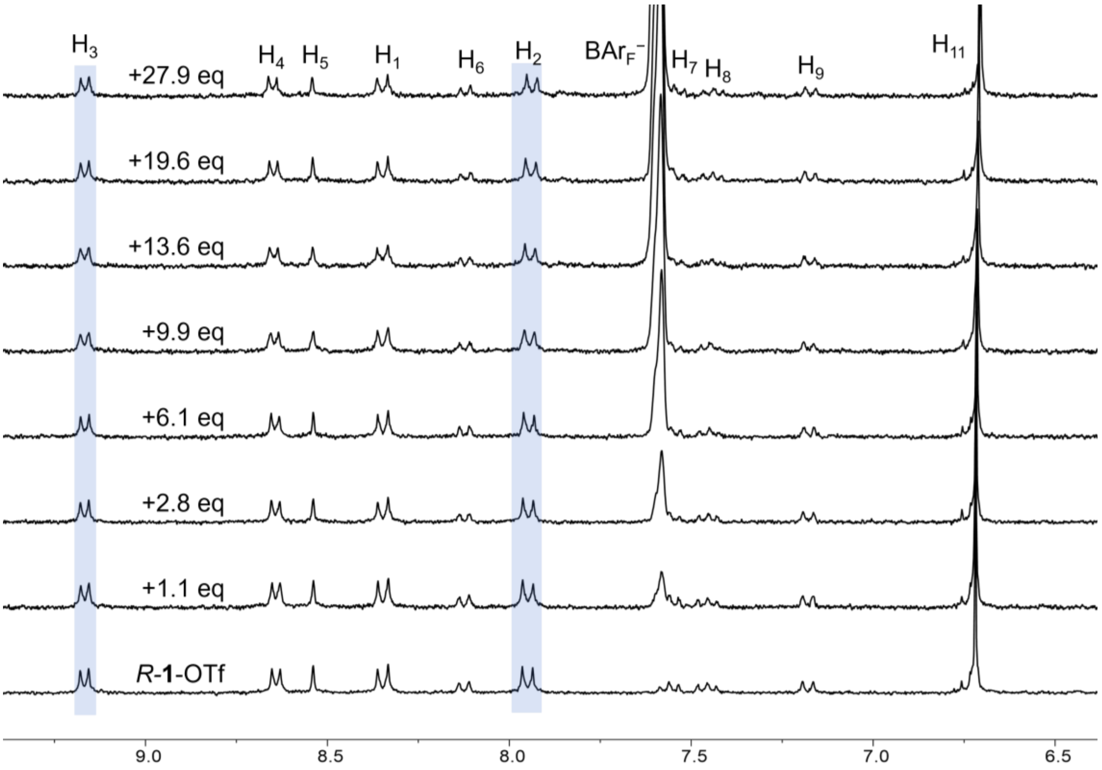

Figure S37. ^{1}H NMR (CD_3OD, 298 K, 400 MHz) titrations of BAr_F^- into a methanol solution of *R*-**1**-OTf (0.30 mM).

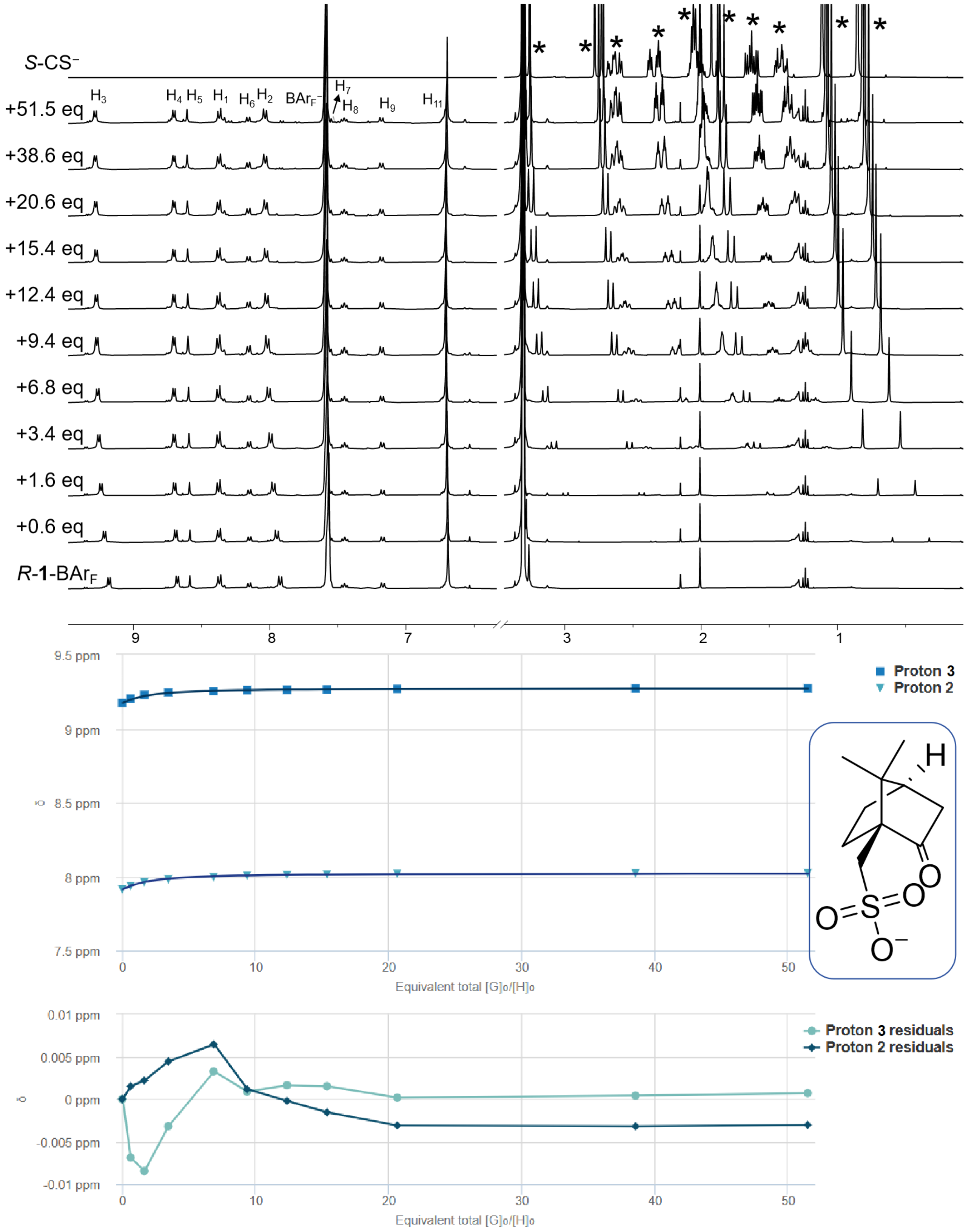

Figure S38. ^{1}H NMR (CD_3OD, 298 K, 400 MHz) titrations of *S*-CS⁻ into a methanol solution of *R*-**1**-BAr_F (0.30 mM) (a) and the corresponding binding isotherms (1:1 system) fitted by BINDFIT (b). A binding constant of $(1.7 \pm 0.2) \times 10^3$ M^{-1} was obtained. The peaks of the guest are marked with asterisks.

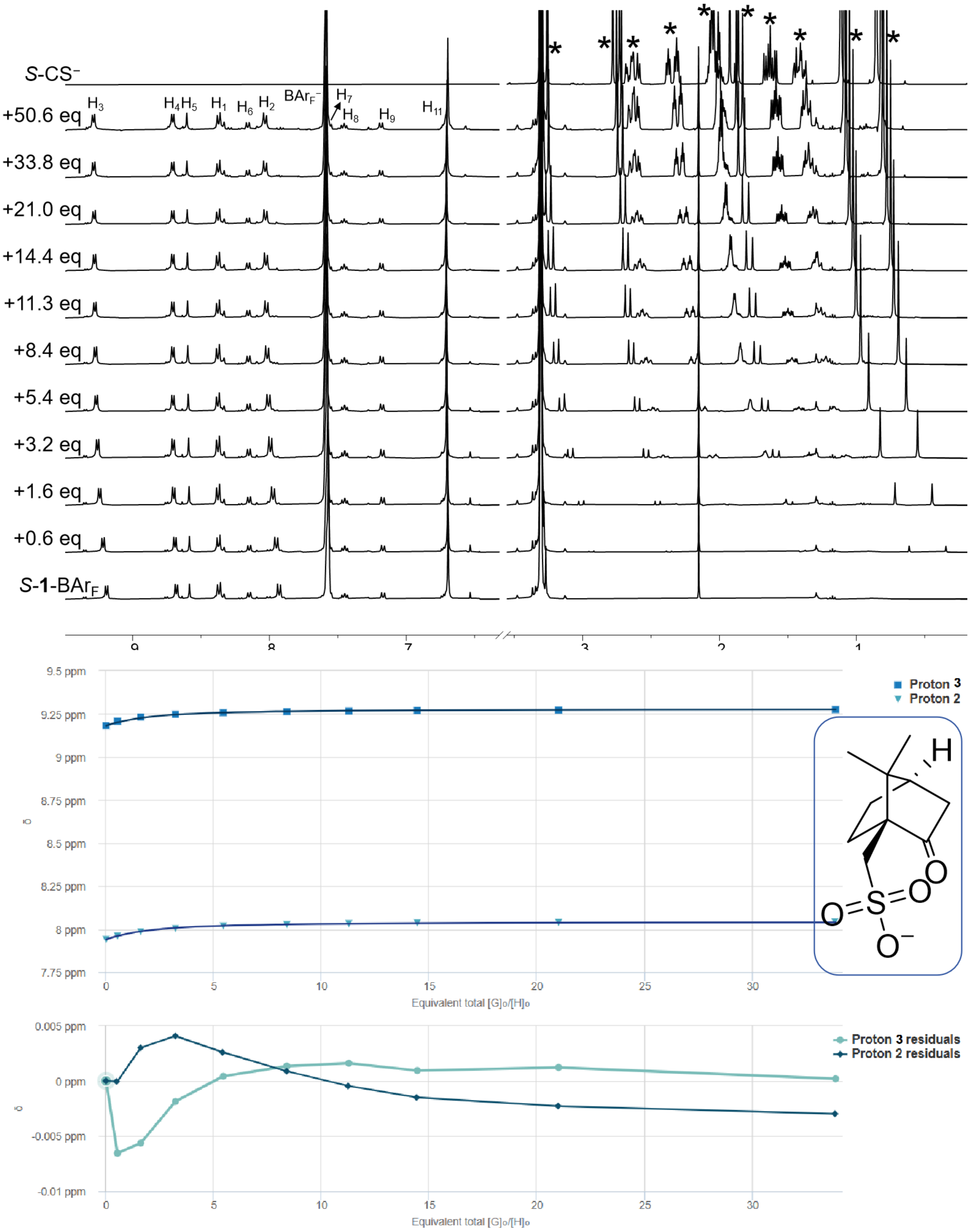

Figure S39. ^{1}H NMR (CD_3OD, 298 K, 400 MHz) titrations of *S*-CS⁻ into a methanol solution of *S*-**1**-BAr_F (0.3 mM) (a) and the corresponding binding isotherms (1:1 system) fitted by BINDFIT (b). A binding constant of $(1.7\pm0.2)\times10^{3}$ M^{-1} was obtained. The peaks of the guest are marked with asterisks.

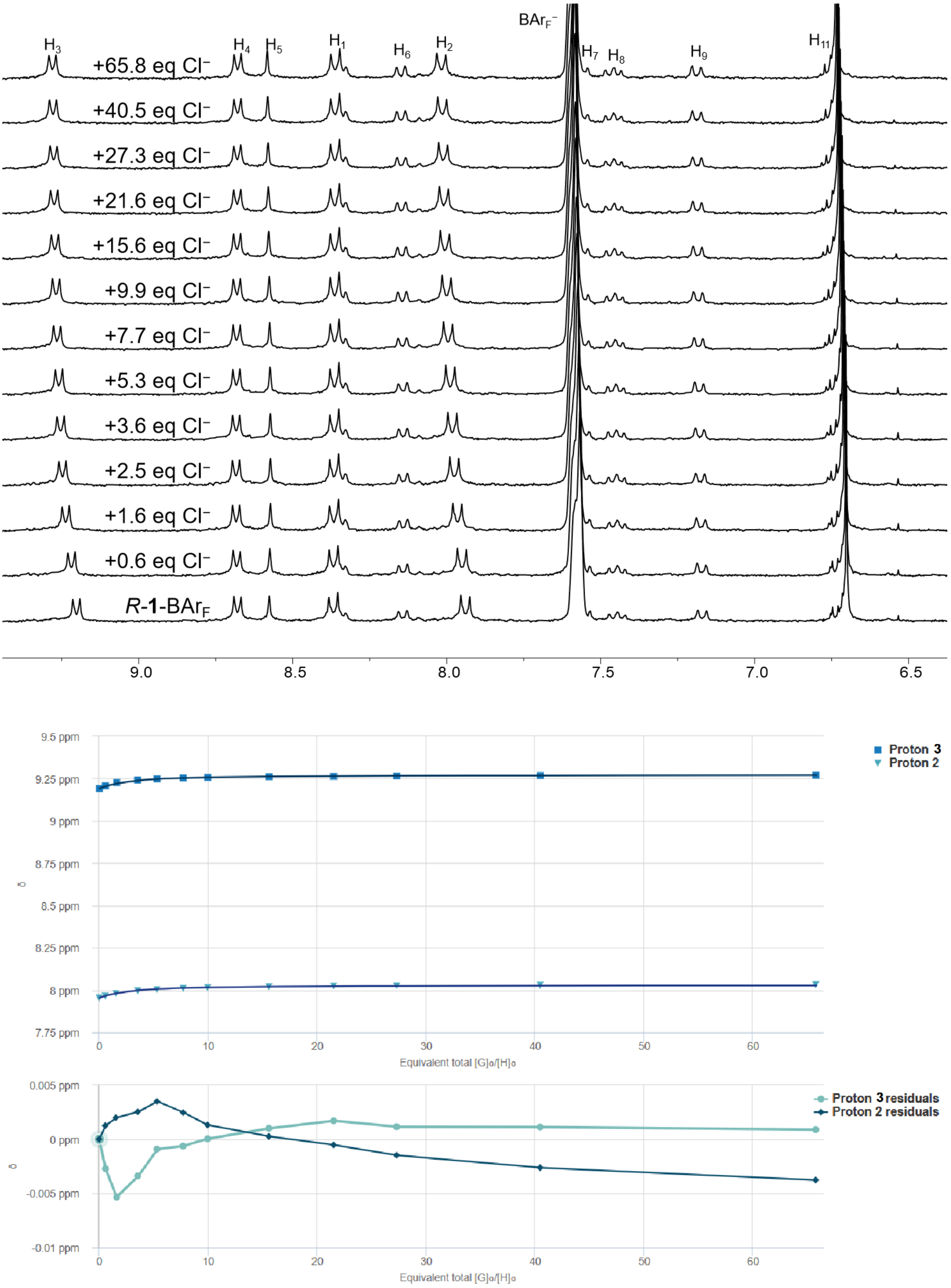

Figure S40. ^{1}H NMR (CD_3OD, 298 K, 400 MHz) titrations of Cl^- into a methanol solution of *R*-**1**-BAr_F (0.3 mM) (a) and the corresponding binding isotherms (1:1 system) fitted by BINDFIT (b). A binding constant of $(5.7\pm0.5)\times10^2$ M^{-1} was obtained.

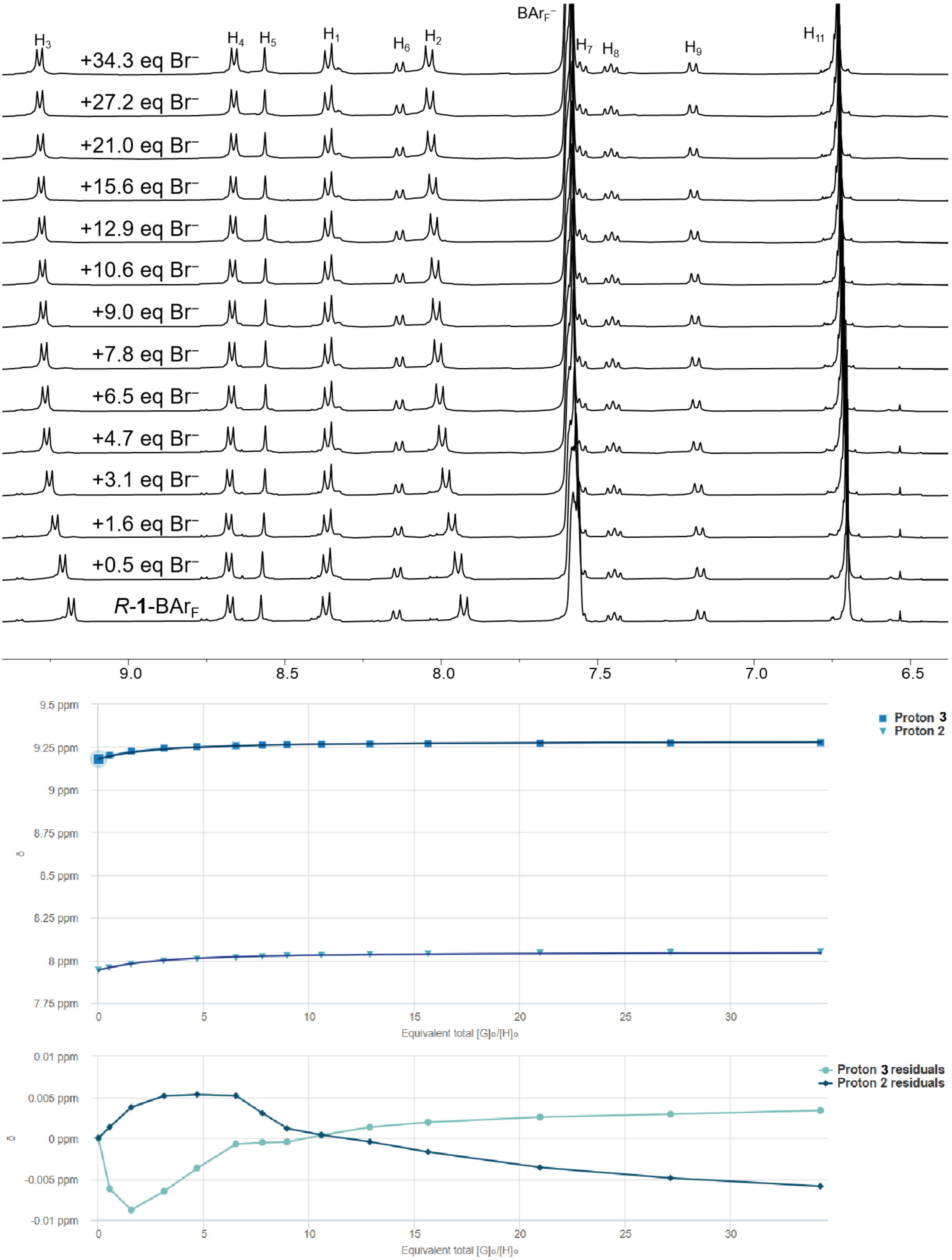

Figure S41. ^{1}H NMR (CD_3OD, 298 K, 400 MHz) titrations of Br^- into a methanol solution of *R*-**1**-BAr_F (0.3 mM) (a) and the corresponding binding isotherms (1:1 system) fitted by BINDFIT (b). A binding constant of $(6.8\pm0.7)\times10^2$ M^{-1} was obtained.

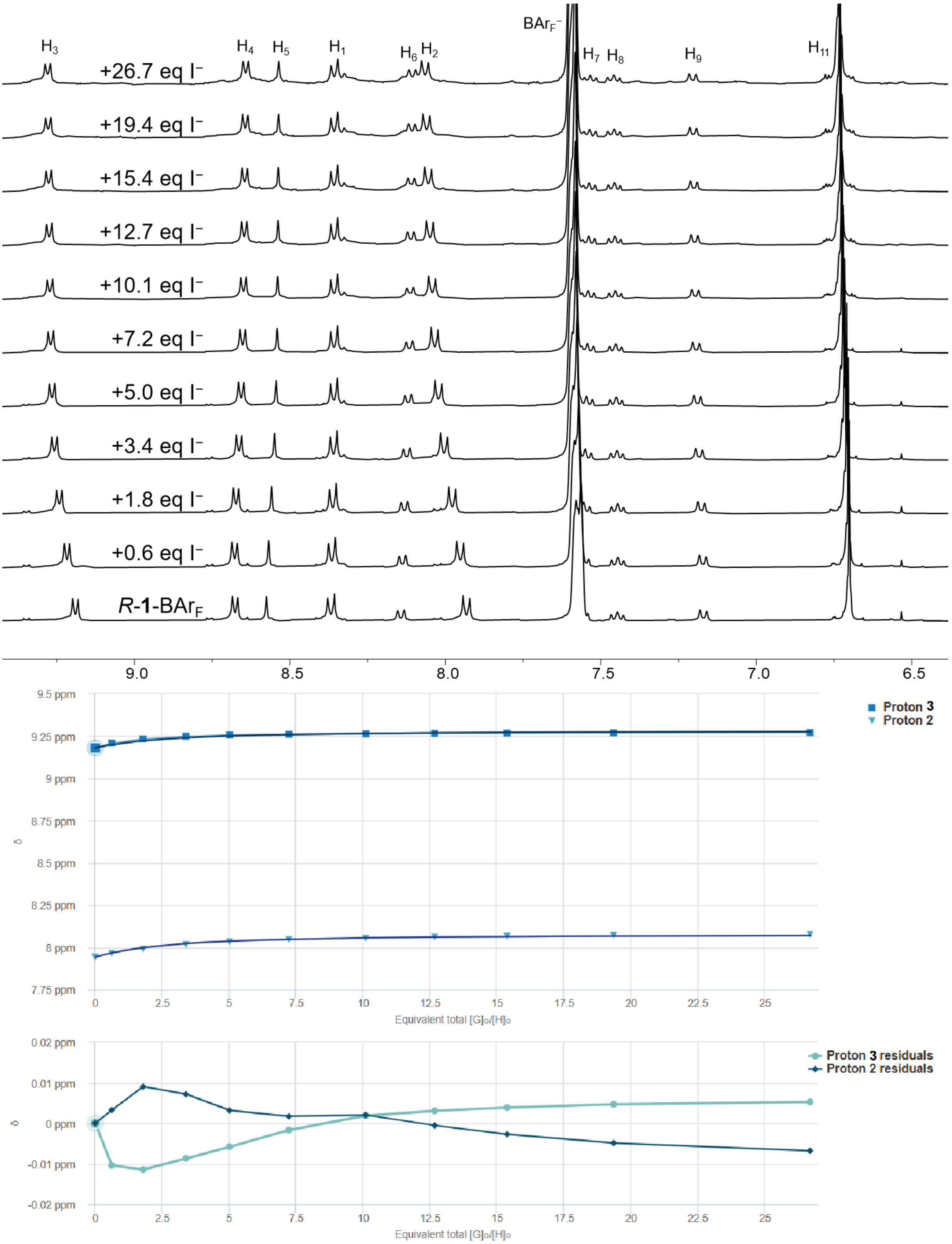

Figure S42.^{1}H NMR (CD_3OD, 298 K, 400 MHz) titrations of I^- into a methanol solution of *R*-**1**-BAr_F (0.3 mM) (a) and the corresponding binding isotherms (1:1 system) fitted by BINDFIT (b). A binding constant of $(6.9\pm0.7)\times10^2$ M^{-1} was obtained.

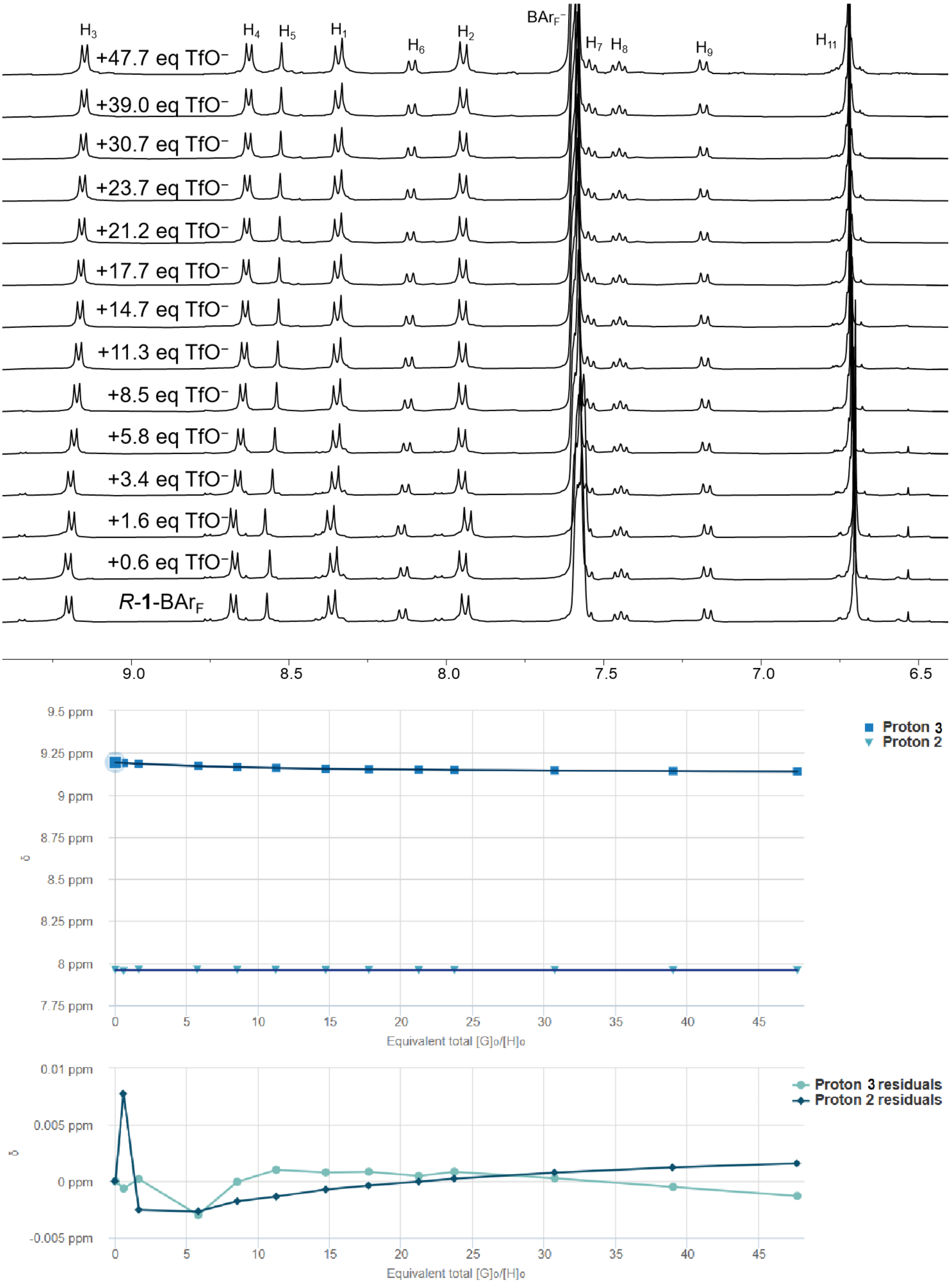

Figure S43. ^{1}H NMR (CD_3OD, 298 K, 400 MHz) titrations of TfO^- into a methanol solution of *R*-**1**-BAr$_F$ (0.3 mM) (a) and the corresponding binding isotherms (1:1 system) fitted by BINDFIT (b). A binding constant of $(1.3\pm0.1)\times10^2$ M^{-1} was obtained.

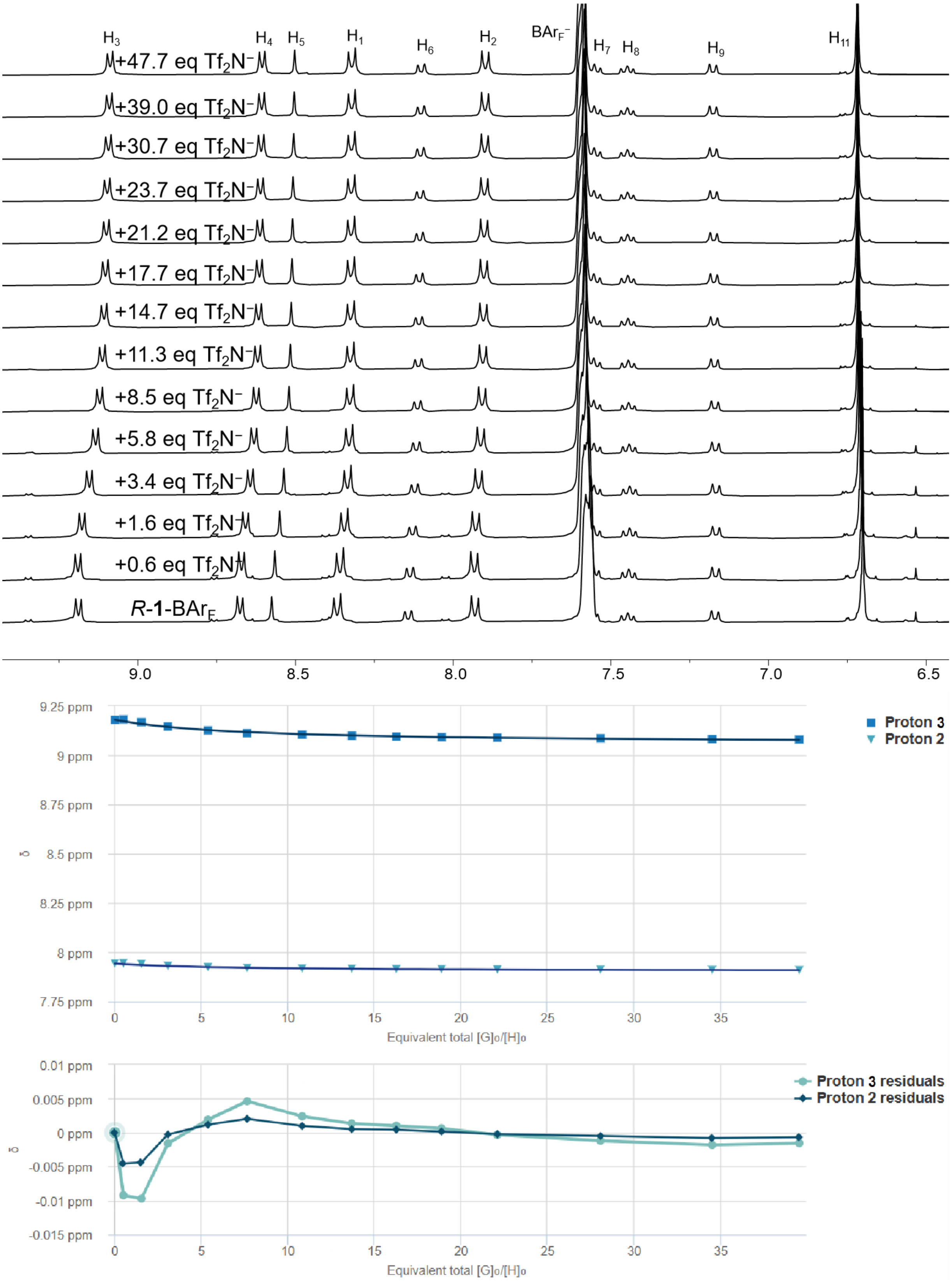

Figure S44. ^{1}H NMR (CD_3OD, 298 K, 400 MHz) titrations of Tf_2N^- into a methanol solution of *R*-**1**-BAr_F (0.3 mM) (a) and the corresponding binding isotherms (1:1 system) fitted by BINDFIT (b). A binding constant of $(2.3\pm0.2)\times10^2$ M^{-1} was obtained.

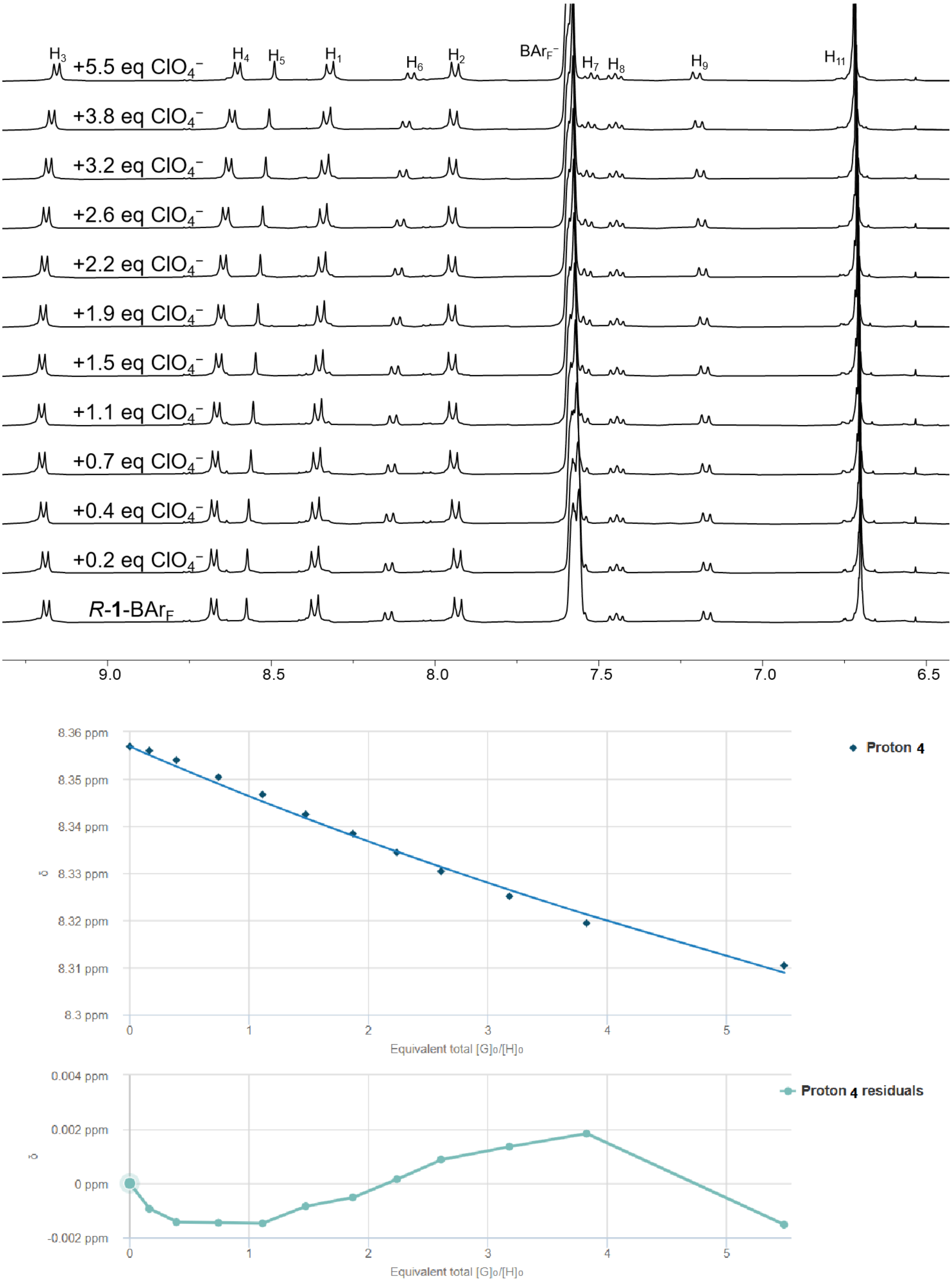

Figure S45. ^{1}H NMR (CD_3OD, 298 K, 400 MHz) titrations of ClO_4^- into a methanol solution of *R*-**1**-BAr_F (0.3 mM) (a) and the corresponding binding isotherms (1:1 system) fitted by BINDFIT (b). A binding constant of $(4.6\pm0.3)\times10^1$ M^{-1} was obtained.

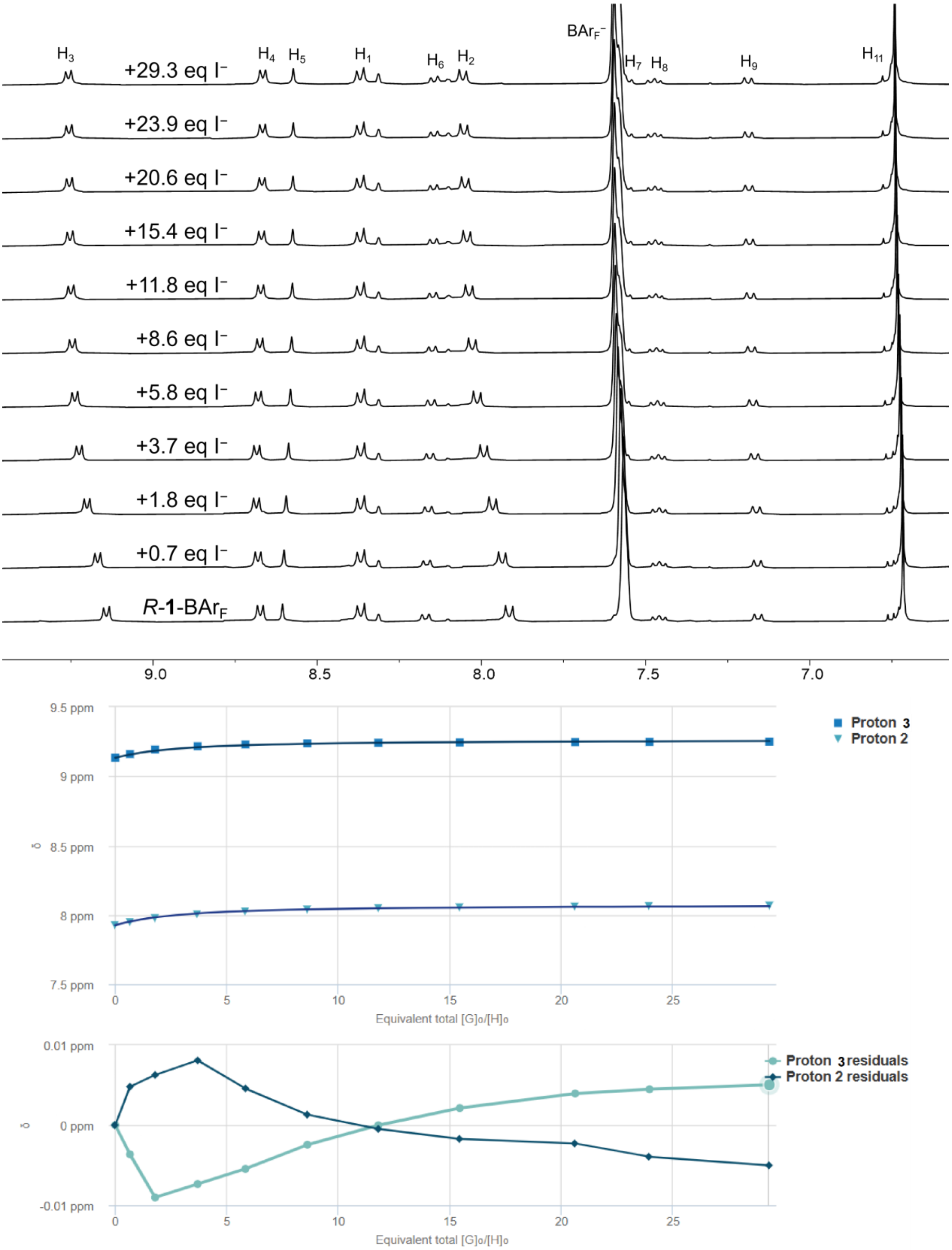

Figure S46. ^{1}H NMR (CD_3OD/D_2O=9:1, 298 K, 400 MHz) titrations of I^- into a solution of *R*-**1**-BAr_F (0.3 mM) (a) and the corresponding binding isotherms (1:1 system) fitted by BINDFIT (b). A binding constant of (7.1±0.7)×10^2 M^{-1} was obtained.

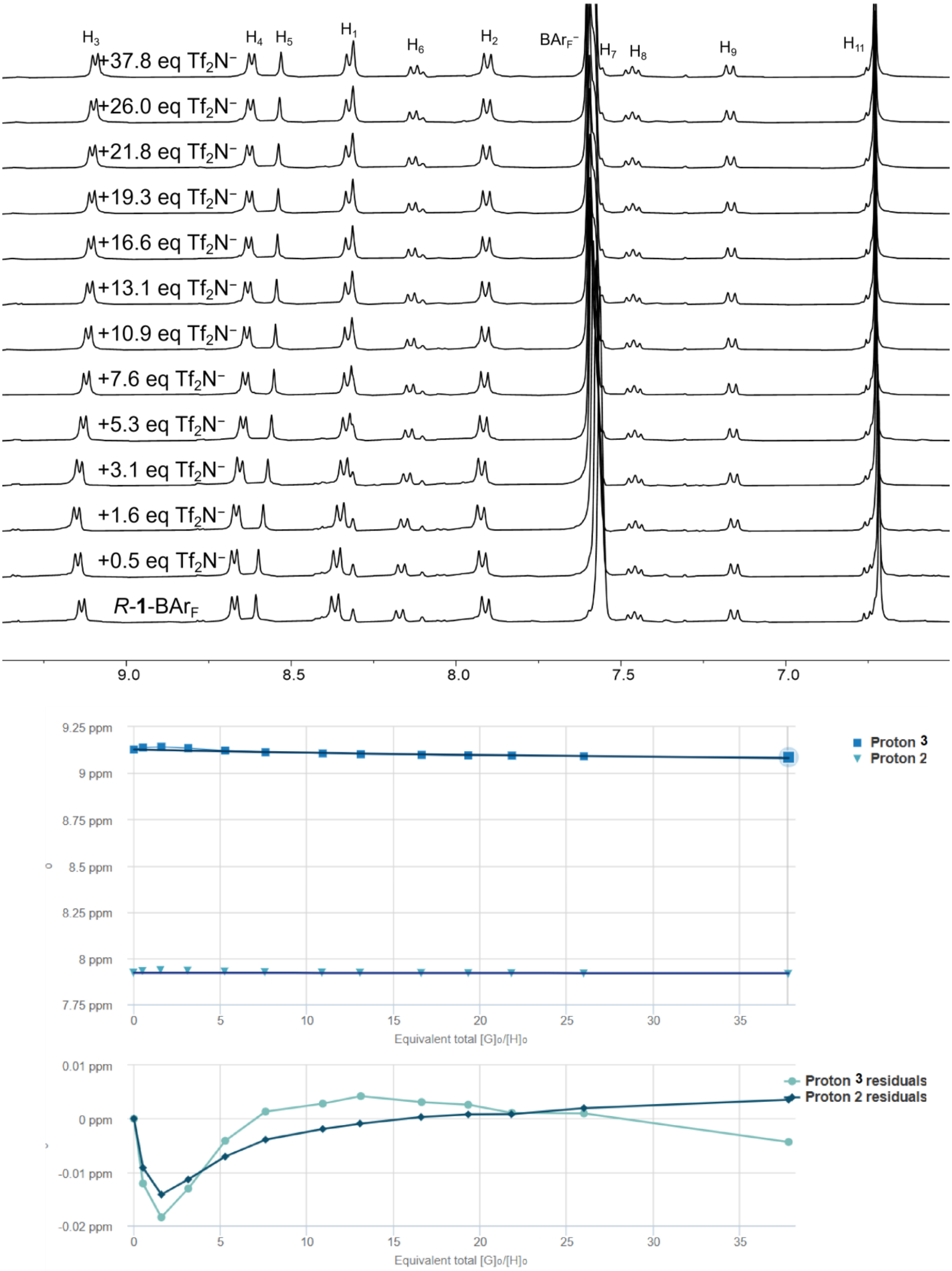

Figure S47.^{1}H NMR (CD_3OD/D_2O=9:1, 298 K, 400 MHz) titrations of Tf_2N^- into a solution of *R*-**1**-BAr$_F$ (0.3 mM) (a) and the corresponding binding isotherms (1:1 system) fitted by BINDFIT (b). A binding constant of (1.7±0.2)×10^2 M^{-1} was obtained.

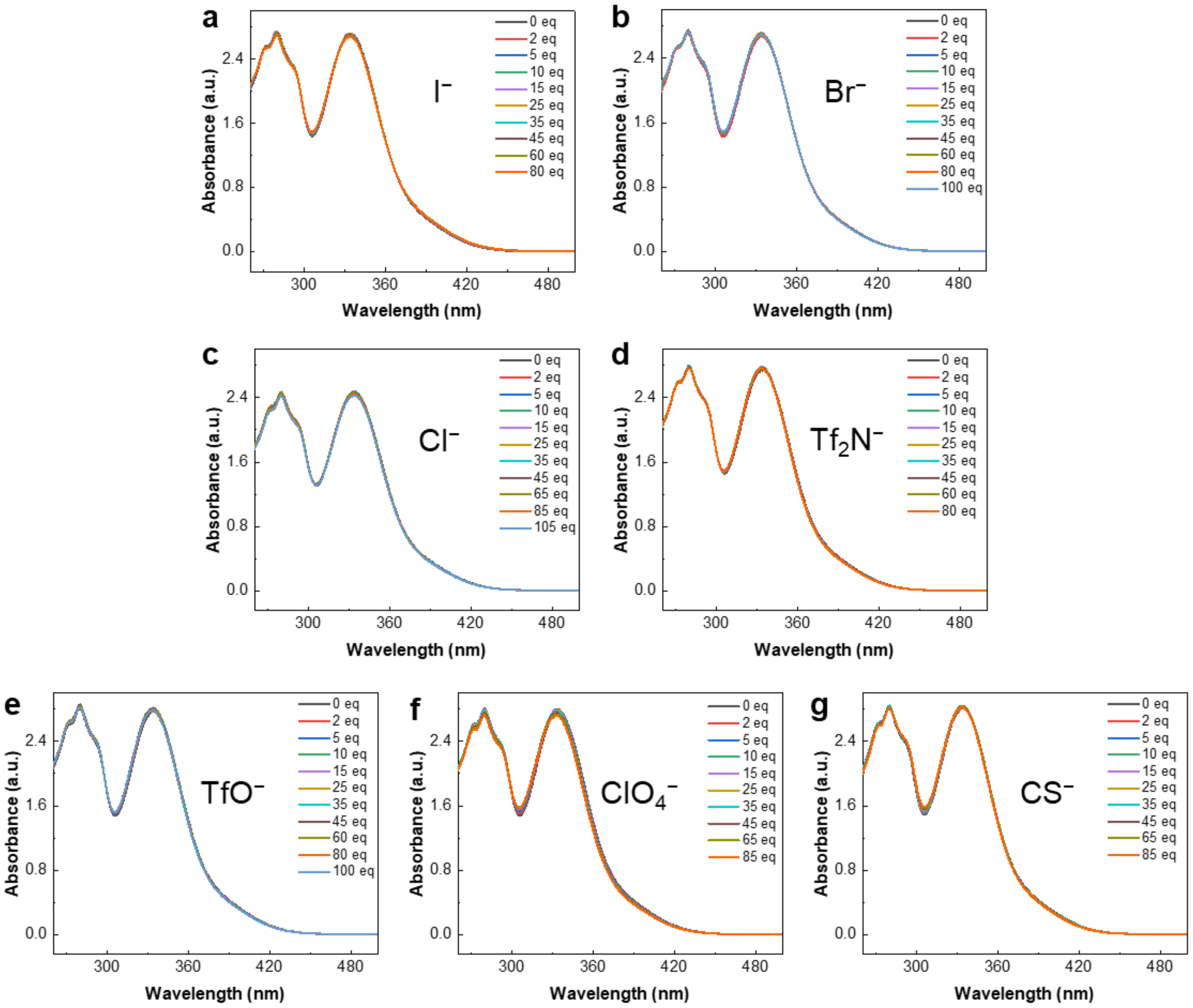

Figure S48. UV-vis spectral titrations of I^- (a), Br^- (b), Cl^- (c), Tf_2N^- (d), TfO^- (e), ClO_4^- (f) and *S*-CS^- (g) into a methanol solution of *R*-**1**-BAr_F (0.014 mM).

3. Aggregation-induced emission

UV-Visible absorption and fluorescence spectra tests were conducted using the same sample. The preparation procedure is as follows: *R*/*S*-**1**-BAr$_F$ (5.0 mg) was dissolved in 4.0 mL of methanol and the solution was stired to obtain a homogeneous stock solution. Then, 400 μL of this stock solution was accurately transferred into ten sample vials. Finally, methanol and ultrapure water were added to each vial in specific volume ratios, creating a test solution system with water volume fractions of 0%, 10%, 20%, ..., 90%, while maintaining a consistent concentration of the coordination cage (1.24x10^{-5} mol/L) in all samples. *R*/*S*-**L**-BAr$_F$ (3.6 mg) was dissolved in 4.0 mL of methanol and the solution was stired to obtain a homogeneous stock solution (3.72x10^{-5} mol/L). Using the same method mentioned above, ten test solutions with the same concentration were prepared. The UV-visible absorption spectra were recorded using a HIMADZU UV-2700 spectrometer, in the 200–800 nm range, with the solution and aggregate samples placed in quartz cuvettes, respectively. The fluorescence spectra were recorded using an RF-6000 spectrometer (Shimadzu, Japan) (λ_{exc} = 365nm), with the solution and aggregate samples placed in quartz cuvettes, respectively.

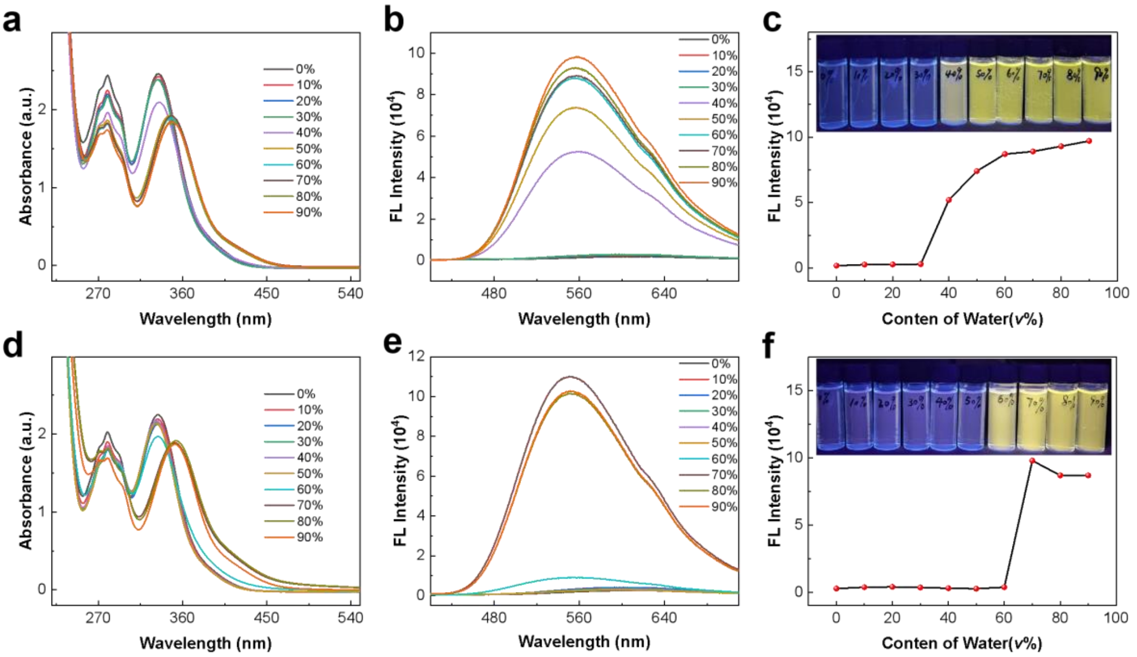

Figure S49. UV–vis absorption spectra (a), fluorescence emission spectra (λ_{exc} = 365 nm) (b), and fluorescence peak intensity (c) of *S*-**1**-BAr$_F$ in MeOH-H_2O mixtures with varying water fractions. Inset of Figure S49c: photographs of the solution at varying water fractions excited at 365 nm. UV–vis absorption spectra (d), fluorescence emission spectra (λ_{exc} = 365 nm) (e), and fluorescence peak intensity (f) of *S*-**L**-BAr$_F$ in MeOH-H_2O mixtures with varying water fractions. Inset of Figure S49f: photographs of the solution at varying water fractions excited at 365 nm.

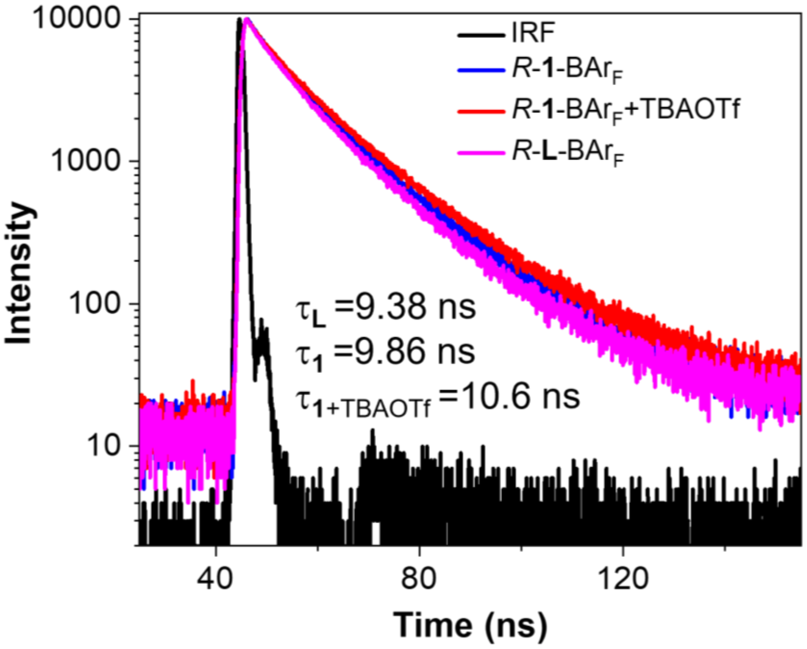

Figure S50. Fluorescence decay profiles of *R*-**1**-BAr$_F$, *R*-**L**-BAr$_F$, and *R*-**1**-BAr$_F$ in the presence of 8 equivalents of TBAOTf in MeOH-H_2O (2:8) mixtures.

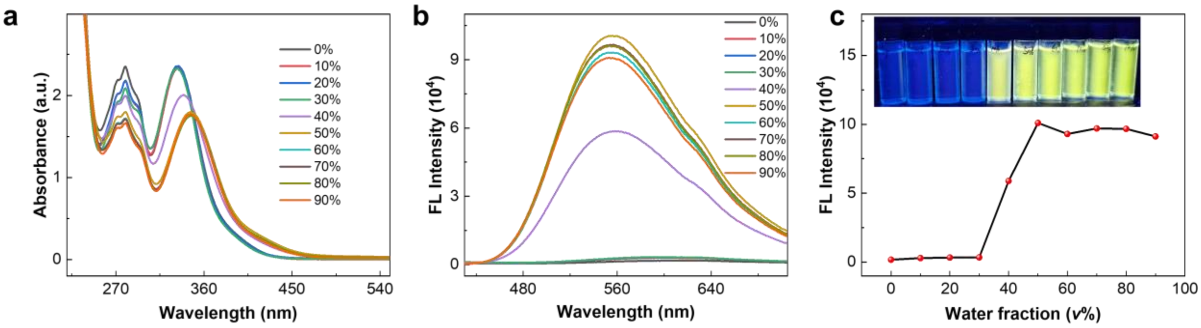

Figure S51. UV–vis absorption spectra (a), fluorescence emission spectra (λ_{exc} = 365 nm) (b), and fluorescence peak intensity (c) of the mixture of *R*-**1**-BAr$_F$ with 8 equivalents of TBAOTf in MeOH-H_2O mixtures with varying water fractions. Inset of Figure S51c: photographs of the solution at varying water fractions excited at 365 nm.

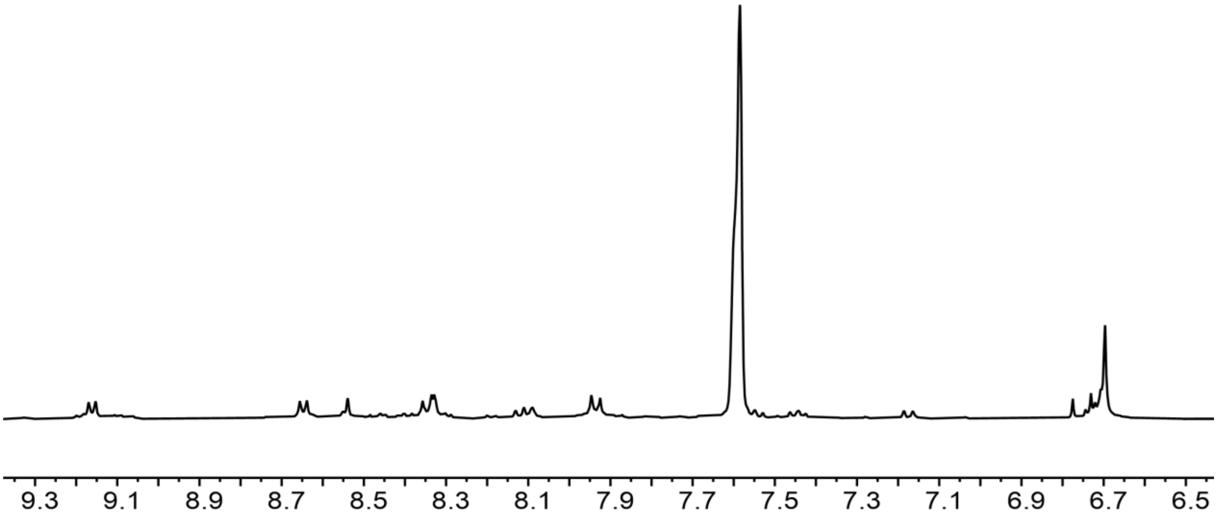

Figure S52. ^{1}H NMR spectrum (CD_3OD, 298 K, 400 MHz) of the aggregates collected from a mixture of *R*-**1**-BAr_F and 8 eq. TBAOTf in a MeOH-H_2O (2:8) mixture.

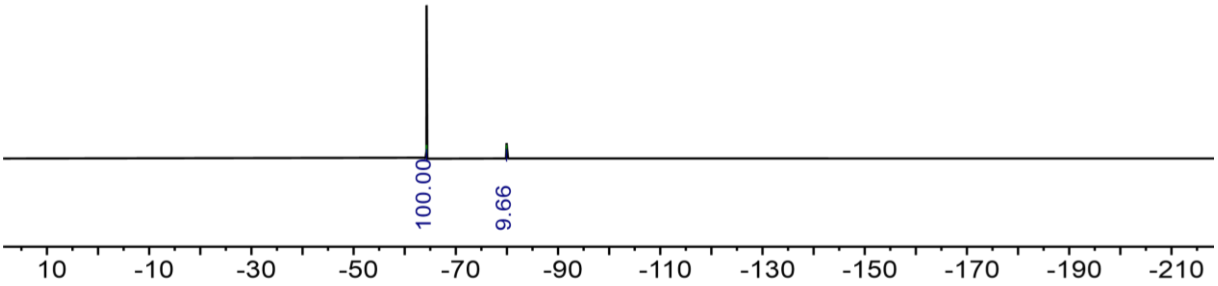

Figure S53. ^{19}F NMR spectrum (CD_3OD, 298 K, 376 MHz) of the aggregates collected from a mixture of *R*-**1**-BAr_F and 8 eq. TBAOTf in a MeOH-H_2O (2:8) mixture.

4. Morphology characterization

The sample was prepared using *R*-**L**-BAr$_F$ or *R*-**1**-BAr$_F$ with a f_w of 80% as described in Section 3. After ultrasonic treatment, the sample was dropped onto silicon pellets, dried, and then analyzed using scanning electron microscope (SEM).

5. Circularly polarized luminescence

Solid *R*/*S*-**L**-BAr_F and *R*/*S*-**1**-BAr_F were used to prepare samples for subsequent CPL measurements. A sample containing 20 mg of *R*/*S*-**1**-BAr_F and 100 mg of KBr was thoroughly ground. The mixture was then pressed into a mold, and testing was performed at room temperature using a JASCO J-1500-150 spectrophotometer (λ_{exc} = 365 nm). Similarly, a mixture of 14.4 mg of solid *R*/*S*-**L**-BAr_F and 100 mg of KBr was ground and pressed into a mold for testing under the same conditions.

6. Computational calculations

6.1 Structure optimization

Structures of *S*-camphorsulfonate, cage *S*-**1** and its 1:1 host–guest complex with *S*-camphorsulfonate were optimized at the PM7 level of theory [5] using the program MOPAC (version 22.0.5) [6] with the default PM7 parameters. Methanol environment was modelled with COSMO implicit solvation model (methanol, ε_r = 32.6) [7].

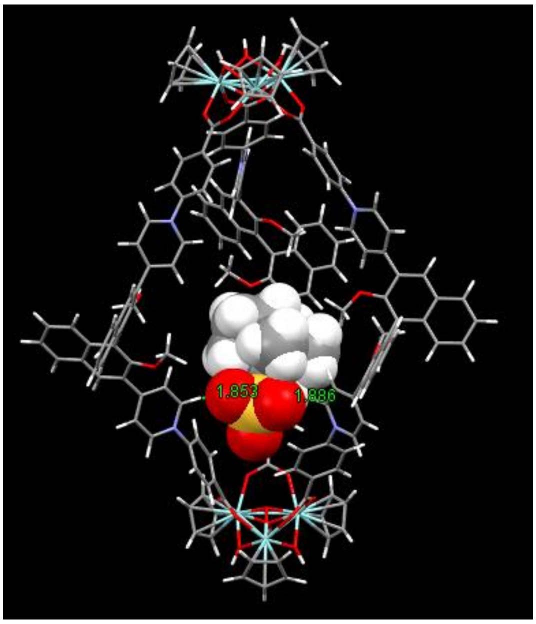

Figure S54. PM7-optimized model of the 1:1 host–guest complex between *S*-**1** and *S*-CS$^-$.

6.2 Volume calculations

In order to determine the available void space within empty cage **1** and the volume of free *S*-camphorsulfonate, MoloVol 1.2.0 [3] calculations were performed on PM7-optimized structures with default parameters and element radii unless otherwise noted. The van der Waals volume of *S*-camphorsulfonate was calculated with MoloVol (grid resolution 0.1 Å). The cavity volume of cage **1** was determined to be *ca.* 708 Å^3 in single probe mode (probe radius: 5.1 Å).

7. References

1. Wu, G.; Bae, Y. J.; Olesinska, M.; Anton-Garcia, D.; Szabo, I.; Rosta, E.; Wasielewski, M. R.; Scherman, O. A., Controlling the Structure and Photophysics of Fluorophore Dimers Using Multiple Cucurbit[8]uril Clampings. *Chem. Sci.* **2020,** *11*, 812-825.
2. http://supramolecular.org/.
3. Maglic, J. B.; Lavendomme, R., MoloVol: an Easy-to-use Program for Analyzing Cavities, Volumes and Surface Areas of Chemical Structures. *J. Appl. Crystallogr.* **2022,** *55*, 1033-1044.
4. Clegg, J. K.; Cremers, J.; Hogben, A. J.; Breiner, B.; Smulders, M. M. J.; Thoburn, J. D.; Nitschke, J. R., A Stimuli Responsive System of Self-assembled Anion-binding $Fe_4L_6^{8+}$ Cages. *Chem. Sci.* **2013,** *4*, 68-76.
5. Stewart, J. J. P., Optimization of Parameters for Semiempirical Methods VI: More Modifications to the NDDO Approximations and Re-optimization of Parameters. *J. Mol. Model.* **2012,** 19, 1-32.
6. http://OpenMOPAC.net.
7. Klamt, A.; Schüürmann, G., COSMO: A New Approach to Dielectric Screening in Solvents With Explicit Expressions for the Screening Energy and Its Gradient. *J. Chem. Soc. Perkin Transactions 2* **1993**, 799-805.
